# A Structural Model of Truncated *Gaussia princeps* Luciferase Elucidating the Crucial Catalytic Function of No.76 Arginine towards Coelenterazine Oxidation

**DOI:** 10.1371/journal.pcbi.1012722

**Published:** 2025-01-21

**Authors:** Nan Wu, Zhi-Chao Xu, Kai-Dong Du, Shen Huang, Naohiro Kobayashi, Yutaka Kuroda, Yan-Hong Bai

**Affiliations:** 1 College of Food and Bioengineering, Zhengzhou University of Light Industry, Zhengzhou, People’s Republic of China; 2 Key Laboratory of Cold Chain Food Processing and Safety Control, Ministry of Education, Zhengzhou University of Light Industry, Zhengzhou, People’s Republic of China; 3 College of Tobacco Science and Engineering, Zhengzhou University of Light Industry, Zhengzhou, People’s Republic of China; 4 RIKEN Center for Biosystems Dynamics Research, RSC, RIKEN, Tsurumi-ku, Yokohama City, Kanagawa, Japan; 5 Department of Biotechnology and Life Science, Graduate School of Engineering, Tokyo University of Agriculture and Technology, Koganei-shi, Tokyo, Japan; University of Maryland School of Pharmacy, UNITED STATES OF AMERICA

## Abstract

*Gaussia* Luciferase (GLuc) is a renowned reporter protein that can catalyze the oxidation of coelenterazine (CTZ) and emit a bright light signal. GLuc comprises two consecutive repeats that form the enzyme body and a central putative catalytic cavity. However, deleting the C-terminal repeat only limited reduces the activity (over 30% residual luminescence intensity detectable), despite being a key part of the cavity. How does the remaining GLuc (tGLuc) catalyze CTZ? To address this question, we built a structural model of tGLuc by removing the C-terminal repeat from the resolved structure of intact GLuc, and verified that the cavity-forming component in GLuc remains stable and provides an open-mouth cavity in tGLuc during 500 ns MD simulations in water. Docking simulation and a followed umbrella sampling analysis further revealed that the cavity on tGLuc has a high affinity for CTZ, with a binding energy of up to -114 kJ/mol. Moreover, R76, a validated activity-critical amino acid residue, resides in the cavity and forms a stable hydrogen bond with CTZ. Then, we constructed a cluster model to examine the CTZ oxidation pathway in the cavity using Density Functional Theory (DFT) calculations. The result showed that the pathway consists of four elementary reactions, with the highest Gibbs energy barrier being 65.4 kJ/mol. Both intramolecular electron transfer and the convergence of S1/S0 potential energy surfaces occurred in the last elementary reaction, which was regarded as the reported Chemically-Initiated-Electron-Exchange-Luminescence (CIEEL) reaction. Geometry and wavefunction analysis on the pathway indicated that R76 plays a vital role in CTZ oxidation, which first anchors the environmental oxygen molecule and induces it to form a singlet biradical state, facilitating its attack on CTZ. Subsequently, R76 and the adjacent Q88, positioned near R76 through the tGLuc refolding process, stabilize the transition states and facilitate the emergence of radical electrons on CTZ at the onset of the CIEEL reaction, which contributes to the subsequent intramolecular electron transfer and the production of excited amide product. This study provides a comprehensive explanation of tGLuc’s catalytic mechanism. However, it is important to note that these findings are specific to tGLuc and may not extend to other CTZ-based luciferases, particularly those lacking arginine in their catalytic cavities, which likely operate via distinct mechanisms.

## Author summary

The luciferase-catalyzed bioluminescence has garnered widespread application across various fields. Among luciferases, *Gaussia* luciferase (GLuc) is distinguished by its small size and intense luminescence, making it an ideal luminescent probe. Despite advances in GLuc’s application development, its catalytic luminescence mechanism remains poorly understood. A major challenge arises from the structural analysis, which reveals GLuc as a monomeric enzyme with a single catalytic chamber. Surprisingly, even after deleting critical C-terminal sequences essential for chamber formation, the truncated enzyme retains considerable catalytic activity. In this study, we utilized molecular dynamics simulations to investigate how the remaining GLuc sequence refolds to regenerate the catalytic chamber. Furthermore, through density functional theory calculations and a cluster model, we mapped the complete reaction potential energy surface for CTZ oxidation within the reformed chamber, identifying R76 as the key catalytic residue and elucidating its role in reducing the reaction barrier to facilitate catalysis. Prior mutagenesis research has confirmed the critical role of R76 in catalytic activity, corroborating our computational results.

## 1. Introduction

**Fig 1:**
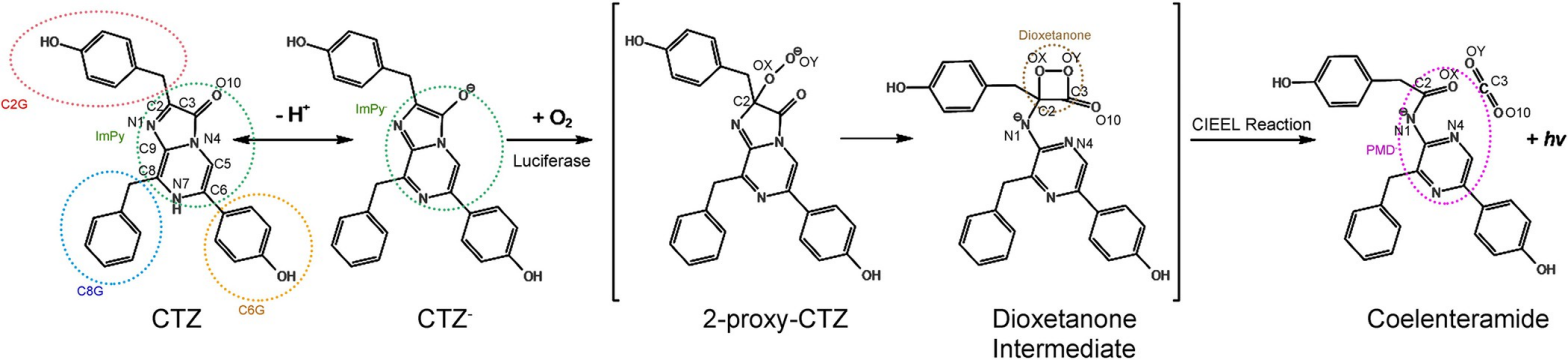
The chemical structure of CTZ and its oxidation pathway catalyzed by luciferase.

Luciferase is a generic term for enzymes that catalyze the oxidation of a substrate, resulting in light emission, which makes them essential biomarkers in bio-imaging applications. Luciferases vary in their origins, structures, and catalytic mechanisms, but the substrates, known as luciferins, are restricted to a few types. Coelenterazine (CTZ) is a common substrate for various marine luciferases (e.g., *Renilla* luciferase [1], *Oplophorus* luciferase [2], etc.), with the core functional group being imidazopyridine (ImPy) at the molecular center. Three aromatic groups are attached to ImPy at its C2, C6, and C8 carbons [3] (abbreviated as C2G, C6G, and C8G in this study). Previous research has shown the oxidation of ImPy involves the N7 deprotonation [4], and the C2 being initially attacked by an oxygen molecule (O_2_) to form 2-peroxy-ImPy. The peroxide rotates around the C2-OX bond to form a C2-OX-OY-C3 four-membered ring. Then the C3-N4 bond breaks and produces an intermediate with dioxetanone. The C2-OX-OY-C3 ring on dioxetanone undergoes a process called Chemically-Initiated-Electron-Exchange-Luminescence (CIEEL) [5] to generate an excited coelenteramide (the ImPy is converted to pyrazinamide, PMD) by releasing a carbon dioxide (CO_2_). The light signal is generated during the relaxation of the coelenteramide [3]. The CTZ-based luciferase (CTZ luciferase) functions to accelerate the reaction process above (Fig 1).

**Fig 2:**
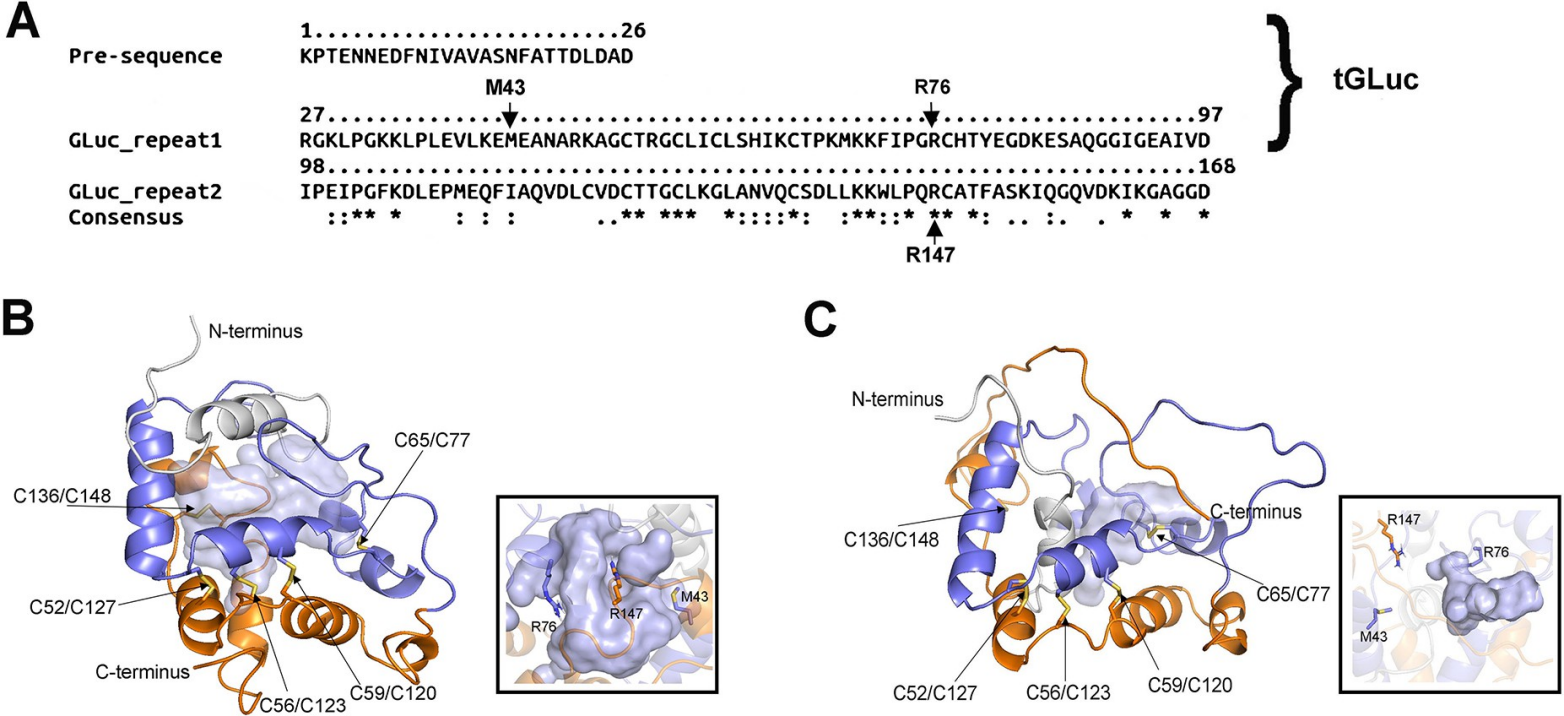
Amino acid sequence and three-dimensional structure of GLuc. (A) The two sequential repeats are located within aa 27–97 and aa 98–168, aligned by CLUSTAL Omega (ver. 1.2.4) [26], with identical residues denoted by “*”, highly similar ones by “:”, and poorly similar ones by “.”. tGLuc comprises the pre-sequence and GLuc_repeat1. (B) and (C) display the AF2 structure (predicted using a local server installed with AF2 [17]) and NMR structure (PDB ID: 7D2O) of GLuc, respectively, with two repeats colored in blue and orange. Five pairs of disulfide bonds are indicated and the internal catalytic cavity is shown by a light blue transparent surface in the structure. The insets in (B) and (C) illustrate the positions of three experimentally identified activity-related residues, M43 [18–21], R76 [10,11], and R147 [11], within the catalytic cavity.

*Gaussia* Luciferase (GLuc), derived from a marine copepod *Gaussia princeps*, belongs to the CTZ luciferase family and is an ideal reporter protein due to its low molecular weight (19.9 kDa) and high luminescence intensity (about 200 times that of RLuc)[6]. However, the catalytic mechanism of GLuc remains elusive. Previous studies showed that GLuc comprises two segments (aa 27–97 and aa 98–168) with similar sequences (Fig 2A), each exhibiting weak luminescence activity independently (about 1% intensity of full-length GLuc) [7]. A Hill plot suggested a cooperation between catalytic centers during GLuc catalysis [8]. Thus, GLuc seems to have two distinct domains, each with a catalytic center [9]. Yet, Kim et al. and Dijkema et al. demonstrated that a single alanine substitution at R76 or R147 (see Fig 2A), two residues respectively in the aa 27–97 repeat and in aa 98–168 repeat, could both abolished GLuc activity [10,11]. This indicates that GLuc’s catalytic centers are not completely independent. Moreover, a recent finding proposed that CTZ luciferase depends on positively charged/protonated amino acid residues (such as His for RLuc [4], Arg for nanoKAZ [12]) in the catalytic cavity to form hydrogen bonds with the negatively charged O10 or N7 atoms on the ImPy of CTZ for substrate recognition (called charge stabilization) [13]. R76 and R147 may have a similar role for GLuc.

Between 2020 and 2021, the solution structure of GLuc determined by NMR [14] (PDB ID: 7D2O) and the predicted structure of GLuc by Alphafold2 (AF2, T1027 in the CASP14) [15–17] were successively reported. The two structures show similarity, with repeats aa 27–97 and aa 98–168 packed antiparallelly with each other and forming the main body of the enzyme that is stabilized by three pairs of disulfide bonds, enclosing a putative catalytic cavity with a positive charge (see Fig 2B and 2C). Differences between the NMR and AF2 structures include: (1) The NMR structure has the N-terminus (aa 1–26) forming an α-helix and inserting into the central cavity, whereas the corresponding sequence in the AF2 structure adheres to the surface of the enzyme body; and (2) The C-terminus (aa 143–168) of the AF2 structure forms an α-helix attached to the enzyme body, while the corresponding sequence in the NMR structure remains in a completely irregular coil state [16]. The folding differences result in a narrow catalytic cavity in the NMR structure, making it difficult to accommodate the complete CTZ, whereas the AF2 structure has sufficient space for CTZ catalysis. Additionally, some reported sites affecting luminescence activity, including M43, which influences luminescence duration [18–21], and the two arginine residues R76 and R147 crucial for luminescence activity [10,11] (Fig 2A), are located within the AF2 structure’s catalytic cavity (Fig 2B, inset); however, in the NMR structure, only R76 locates within the catalytic cavity. M43 and R147 is gapped with the cavity by a low conserved N-terminal α-helix (I11-N17, Figs 2C inset and S1), therefore it is difficult to understand the importance of these two residues towards activity by using the NMR structure. We speculated that conformational transitions [22] may occur on GLuc in solution, the AF2 structure is more likely to be the conformation of tGLuc during CTZ catalysis, with aa 1–97 and aa 98–168 fold into two hemispherical structures, enclosing a larger open cavity for substrate binding and catalysis (most of the reported structure of CTZ luciferases have relatively spacious catalytic cavities, such as nanoKAZ [23], aequorin [24], obelin [25], etc.). In contrast, the NMR structure likely represents tGLuc’s conformation when not performing catalysis, with a smaller catalytic cavity that benefits to structural stability in solution.

The AF2 structure exhibits a single-domain and single-cavity architecture, which appears to be inconsistent with the finding that GLuc has two catalytic centers. However, given the strong interdependence of the active centers, we hypothesize that the repeats aa 27–97 and aa 98–168, each containing an active center, are located within the same central cavity and interact with each other. This may account for the far superior of GLuc to that of some luciferases with a single active center (RLuc [27], OLuc [23], aequorin [24], etc.). Nevertheless, the elucidation of GLuc’s catalytic mechanism remains challenging, as the number of CTZ molecules bound in the cavity (one or two) and the cooperative mechanism are still elusive. In 2016, Hunt reported a truncated GLuc (tGLuc) consisting of the aa 1–97 fragment that includes the pre-sequence (aa 1–26) and the aa 27–97 repeat, showing a luminescence intensity reaching 30% of full-length GLuc, with a nearly identical luminescent spectrum [28]. This suggests that tGLuc likely preserves the core structural framework of GLuc’s catalytic center within the aa 27–97 repeat. As tGLuc eliminates interference from the active center within aa 98–168, elucidating the chemical mechanism behind tGLuc’s catalysis of CTZ oxidation becomes more feasible than for full-length GLuc.

Therefore, this investigation targets tGLuc, with the aim of using computational methods to reveal the mechanistic interplay between GLuc’s active center within the aa 1–97 fragment and the substrate. Initially, the tGLuc structure model was obtained by removing the aa 98–168 repeat from the AF2 structure, followed by a Molecular Dynamics (MD) simulation, ensuring the achievement of structural stability. Subsequently, the tGLuc structure underwent docking with CTZ, followed by another MD simulation until the stability of the docked complex was established. Density Functional Theory (DFT) is a useful tool for probing the chemical mechanisms of enzyme-catalyzed substrate reactions. In recent years, numerous theoretical studies using DFT methods have explored the luciferin oxidation mechanism [29–31]. Here, we extracted the active center region from the tGLuc-CTZ complex and built a cluster model that enclosed an O_2_, followed by identifying all the CTZ oxidation transition states and intermediates in the catalytic cavity through DFT calculations. As the result, a CTZ oxidation pathway that involves a CIEEL reaction [29,30] was identified, which displays the intra-molecular electron transfer within oxidized CTZ and S0/S1 Potential Energy Surface (PES) convergence. We performed the analysis on geometries and wavefunctions on the pathway, which indicates that R76 is the key catalytic residue, as it facilitates the O_2_ addition to CTZ, stabilizes the transition state of the oxidized CTZ and promotes the CIEEL reaction.

## 2. Theoretical method

### 2.1 Software

GROMACS (Ver. 2021.2) [32] were employed for performing MD simulation; GaussView6 (Ver. 6.1.1) [33] was used for modeling CTZ, whose force filed was generated using sobtop (Ver. 3.1) [34]. Molecular docking was performed using AutoDock (Ver. 4.2) [35]. Quantum chemical calculations were performed using xtb (Ver. 6.5.1) [36] and Gaussian16 (Ver. C.01) [37], with subsequent wavefunction analysis conducted using Multiwfn (Ver. 3.8) [38,39]. All molecular structures were visualized using PyMOL [40] or VMD [41].

### 2.2 Computational details

#### 2.2.1 Structure modeling of tGLuc and CTZ

The initial tGLuc model was constructed by manually excising the aa 98–168 repeat of AF2 structure (coordinates file was provided in the S1 Supplemental Structures, Intact_GLuc_AF2.pdb), and placed in a periodic cubic box with a minimum distance of 1 nm between the protein surface and the box edges, using the Amber14SB force field [42]. The box was then solvated with SPC216 water models (using the TIP3P force field) and Na^+^/Cl^-^ ions up to 100 mM to mimic the salt solution that favored by GLuc catalysis [9,43,44] and neutralize the system. The system was energy-minimized by the steepest-descent and conjugate-gradient methods, until the maximum force was below 100 kJ/mol/nm. The system was then equilibrated by a 100 ps NVT ensemble (constant number of particles, volume, and temperature) followed by a 100 ps NPT ensemble (constant number of particles, pressure, and temperature), maintaining the temperature at 300 K and the pressure at 1 bar. After equilibration, a 500 ns all-atom MD simulation was performed with a 2 fs time step. During the simulation, the hydrogen bonds were constrained by the LINCS algorithm [45], the long-range electrostatic forces were calculated by the PME method [46] with a 1.4 nm cutoff radius, and the temperature and pressure coupling were achieved by the Velocity-rescale [47] and Parrinello-Rahman [48] methods, respectively. The energy and trajectory data were recorded every 10 ps. After the simulation, a Principal Component Analysis (PCA) was performed on the 500 ns trajectory. The eigenvalues were obtained by considering all atoms of the tGLuc, and the 1st (PC1) and 2nd (PC2) eigenvectors were used to generate a 2-dimensional energy landscape. A representative frame with the lowest energy was selected from this landscape.

The structure of deprotonated CTZ (used for subsequent molecular docking and MD simulations) was modeled by GaussView6 and optimized by Gaussian16 at the ωB97XD/6–311+G(d,p) level of theory [49,50]. The wavefunction of the modeled CTZ was analyzed by Multiwfn to compute the Restrained Electro Static Potential (RESP) atomic charges [51], which along with the optimized structure of CTZ were input to sobtop to generate the Generation Amber Force Field (GAFF) force field [52].

#### 2.2.2 Docking of tGLuc with CTZ and construction of the O_2_-containing active_cluster model

The representative structure of tGLuc was docked with CTZ using Autodock 4.2 in a semi-flexible manner, where tGLuc was rigid and CTZ was flexible, and the docking was restricted to the concave surfaces on the molecular exterior. Based on binding energy analysis and the reported luciferase catalysis mechanism, the optimal conformation of docked complex was selected and subjected to 100 ns MD simulations under the same conditions as in section 2.2.1. Then, PCA was performed on the 100 ns trajectory using all atoms of the tGLuc-CTZ complex, and the lowest-energy frame was selected for calculating the binding energy and constructing the O_2_-containing active_cluster model.

The tGLuc-CTZ conformation was extracted from the selected frame as the representative structure and then performed Umbrella Sampling (US) simulations on the CTZ within it. Firstly, the representative tGLuc-CTZ conformation were aligned parallel to the z-axis. A periodic cubic box, centered on the complex, was constructed, ensuring the distance between the complex and the box exceeded 1 nm. The simulation box was extended along the z-axis, with dimensions of 6.7 nm, 6.7 nm, and 9.0 nm. As described in method 2.2.1, the box was filled with solvent and ions, followed by energy minimization, and NVT and NPT equilibration. Subsequently, the ligand was extracted from the tGLuc pocket into the solvent over 300 ps at a rate of 0.01 nm per ps using a force of 5000 kJ/mol/nm. Snapshots were taken at each ps, yielding a total of 300 configurations. From these, 40 configurations were selected as starting points, each subjected to 100 ps NPT equilibration followed by a 10 ns MD run. The Potential of Mean Force (PMF) was calculated using the Weighted Histogram Analysis Method (WHAM) in GROMACS.

Then, the selected lowest-energy frame was used to construct the O_2_-containing active_cluster mode. Ten random water molecules in this frame were replaced with O_2_, which used the Transferable Potentials for Phase Equilibria (TraPPE) force field [53] with negatively charged oxygen atoms and a positive dummy atom at the center, better representing O_2_ than GAFF. The system with O_2_ was simulated for 50 ns under the same conditions as in section 2.2.1, except for a reduced time step of 1 fs. During the 50 ns simulation, an O_2_ penetrated the cavity twice and remained stable in close proximity to the C2 of ImPy (the oxidation site, as depicted in Fig 1) for over 4 ns, allowing for the oxidation process to take place. Consequently, a frame with the minimum O_2_-C2 distance was selected from the 50 ns trajectory, and from this frame, the atomic entities within a 3.5 Å radius of CTZ, including the O_2_, water, and amino acid residues (all side-chain atoms and the CTZ contacting main-chain atoms were retained, while the C_α_ were saturated with hydrogens), were isolated to establish an active_cluster model. The model was then followed with a geometry optimization using GFN2-xTB [54]. This methodology was previously applied successfully to model the active_cluster of nanoKAZ, another CTZ luciferase, in our prior work [12].

#### 2.2.3 DFT calculation and wavefunction analysis

The active_cluster was performed geometry optimization, (rigid) scanning, transition state (TS) searching, and intrinsic reaction coordinate (IRC) [55] calculation using the DFT method on S0 basic state with a mixed basis set approach in Gaussian16. ωB97XD, a long-range-corrected hybrid functional that has been effective in elucidating the oxidation mechanisms of various luciferins [31,56,57], was used as the theoretical method. The open-shell (U) approach and the broken-symmetry technique that mixes the HOMO and LUMO to generate an initial biradical guess were applied with the ωB97XD. The stability of all generated wavefunctions for the stationary points on the S0 PES were verified, and a frequency analysis confirmed the absence of imaginary frequency for the intermediate (Int), and only one imaginary frequency for TS. The S1 exited state was computed by a Time-Dependent DFT approach (TD-ωB97XD) [58]. The mixed basis set strategy used above was: atoms with electron transfer tendency, including the ImPy of CTZ, O_2_, guanidine group of R76, acylamino group of Q88, used the 6-31G(d,p) basis set [59], while other atoms used 3-21G*[60] (see S2 Fig).

The electronic energy of each S0 PES stationary point was reevaluated under UωB97XD with all atoms using the 6-31G+(d,p) basis set, and the reevaluated electronic energy was added by the thermal correction at 298.15 K and 1 atm that obtained during frequency analysis to calculate Gibbs energy. Furthermore, the oxygenated CTZ and surrounding amino acid residues in above stationary points were extracted from the active_cluster and calculated wavefunctions separately. All wavefunctions at the UωB97XD/ 6-31G+(d,p) level were analyzed by Multiwfn for: (1) computing the Electron Density (ED) and Electron Spin Density (ESD) for the active_cluster (ED^cluster^/ESD^cluster^), proxy-CTZ (ED^CTZ^/ESD^CTZ^), and surrounding amino acid residues (ED^res^/ESD^res^), individually. The Electron Density Difference (EDD) was calculated by: ED^cluster^—ED^CTZ^—ED^res^; (2) investigating the intermolecular non-covalent interactions between the oxygenated CTZ and surrounding amino acid residues by the Independent Gradient Model based on the Hirshfeld partition (IGMH) method [61].

## 3. Result and discussion

### 3.1 The stable folded structure and the putative catalytic cavity of tGLuc

**Fig 3:**
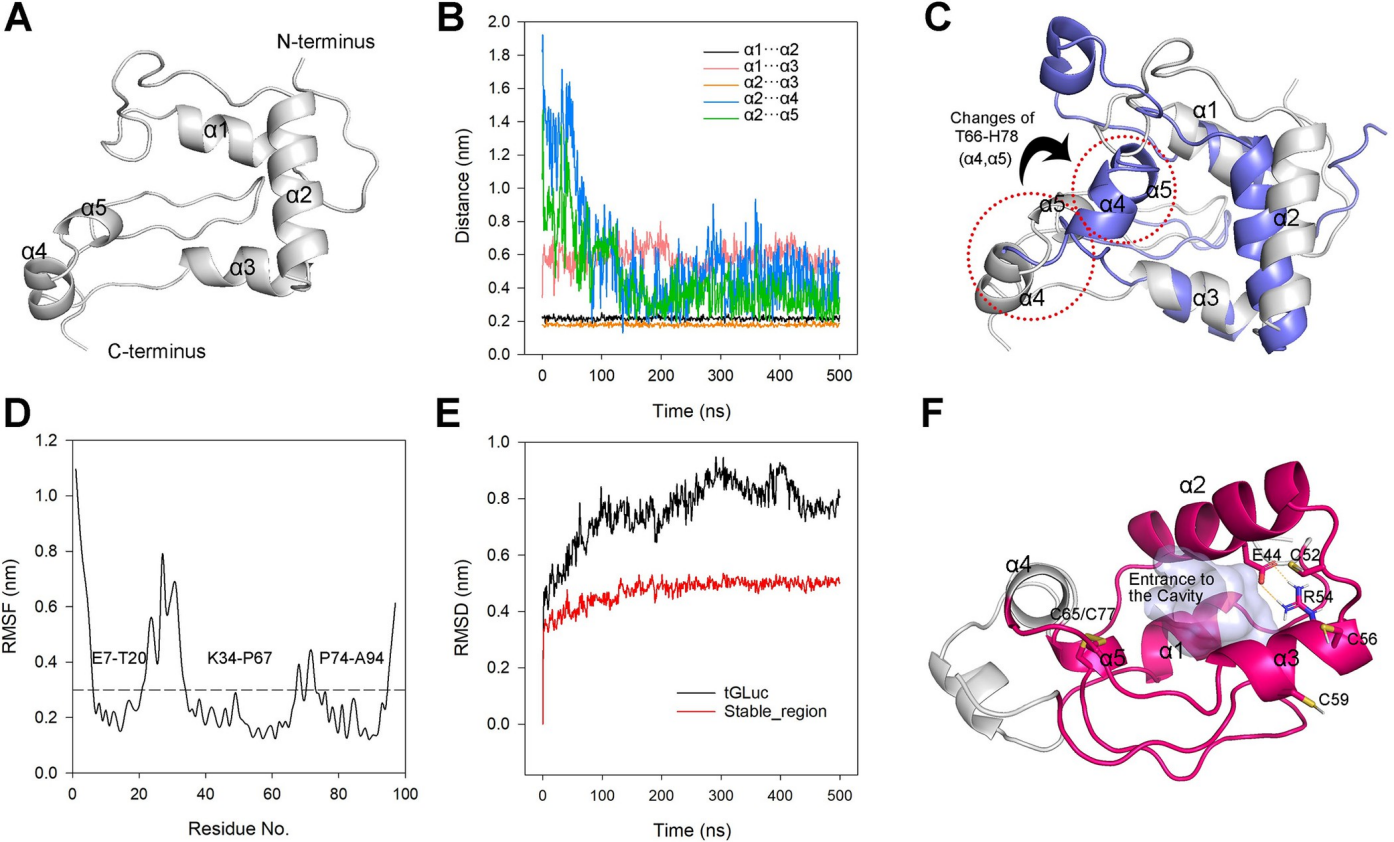
500 ns MD simulation and the representative structure of tGLuc. (A) Initial tGLuc structure isolated from the AF2 model, comprising five α-helices; (B) Time evolution of the distances between the centroids of each α-helix during the MD simulation; (C) Superposition of tGLuc structures before (white) and after (blue, PCA-identified representative structure) the 500 ns MD simulation, with the T66-H78 fragment highlighted by a red dashed circle; (D) RMSF of all tGLuc residues, indicating that the E7-T20, K34-P67, and P74-A94 regions exhibit RMSF values below 0.3 nm, marking them as stable_region; (E) RMSD of the entire tGLuc (black) and the stable_region (red); (F) Representative refolded structure identified by PCA analysis, with the stable_region highlighted in red and the putative catalytic cavity displayed in transparent light blue.

The initial tGLuc structure, comprising five α-helices (α1: I11-A19; α2: L37-A50; α3: R54-H62; α4: P67-F72; α5: P74-C77), was derived by excising the aa 98–168 repeat from our previously predicted intact GLuc structure using AlphaFold2 [16,17] (Fig 3A), followed by a 500 ns MD simulation in an explicit solvent environment. During simulation, α1 and α3, initially adjacent to α2, maintained a consistent distance of approximately 0.2 nm relative to α2; the distance between α1 and α3, flanking a long loop turn, also remained stable at around 0.6 nm. In contrast, α4 and α5 rapidly approached α2 before 200ns, and ultimately stabilizing at a distance of approximately 0.4 nm (Fig 3B). To verify the reproducibility of the MD simulation, we conducted two additional independent 500 ns simulations with different initial velocities. The results showed that α1, α2, α3, and α5 maintained consistent relative distance with the initial simulation. However, in the second simulation, α4 exhibited higher mobility, with its distance from α2 remaining around 0.8 nm for an extended period before shortening to 0.5 nm just before the end of simulation (S4 Fig). To visualize these structural changes, we used PCA to analyze the simulation trajectory and extract a representative tGLuc refolded structure (In the manuscript, we only display and utilize the representative structure from the initial simulation, where all amino acid residues are within the reasonable regions of the Ramachandran plot, see S3A Fig; the representative structures from the subsequent two simulations are shown in S4 Fig, with high similarity among the selected representative structures from all three simulations). We superimposed the refolded representative structure of tGLuc with the AF2 structure aa 1–97 (the initial structure prior to simulation), revealing high similarity between the refolded and initial structures in the α1-α3 region. The T66-H78 segment, stabilized by a disulfide bond pair (C65/C77), formed a fixed structural element containing α4 and α5 that converged toward the center of tGLuc, creating a distinct open-mouthed cavity (Fig 3C). As previously mentioned, the relatively stable α1-α3 and α5 helices across the three simulations formed the main body of this cavity, while the highly mobile α4 was located at the cavity mouth. We hypothesize that α4 may function as a lid regulating the opening and closing of the cavity. Furthermore, the inward movement of the T66-H78 region toward the center appears to be a unique mechanism for tGLuc cavity formation, as no significant structural changes were observed in this region during MD simulations of the full-length GLuc (S5 Fig).

Subsequent analysis of the Root Mean Square Fluctuation (RMSF) across individual amino acid residues delineated three regions of stability within tGLuc: E7-T20, K34-P67, and P74-A94, each exhibiting RMSF values below 0.3 nm, collectively termed the stable_region (Fig 3D). Additional scrutiny involving RMSD (Fig 3E), Radius of gyration (Rg, S3B Fig), and Solvent Accessible Surface Area (SASA, S3C Fig) indicated minimal conformational deviation within the stable_region post 100 ns, in contrast to the fluctuating entire tGLuc. The cavity forming fragments α1-α3 and α5 were all locating in the stable_region, and the representative structure indicated the stable_region was stabilized by the disulfide bond C65/C77 and an ionic bond E44/R54. Moreover, although our earlier research indicated that C52, C56, and C59 in full-length GLuc should respectively form bonds with C127, C123, and C120 within the aa 98–168 repeat[14], recent studies suggest the possibility of intra-repeat disulfide bond formation between C56 and C59[11]. Therefore, C52, C56, and C59, that are proximal to each other in tGLuc are highly likely to form one stable disulfide bond.

### 3.2 Molecular Docking and Binding Energy Analysis of tGLuc with CTZ^-^

**Fig 4:**
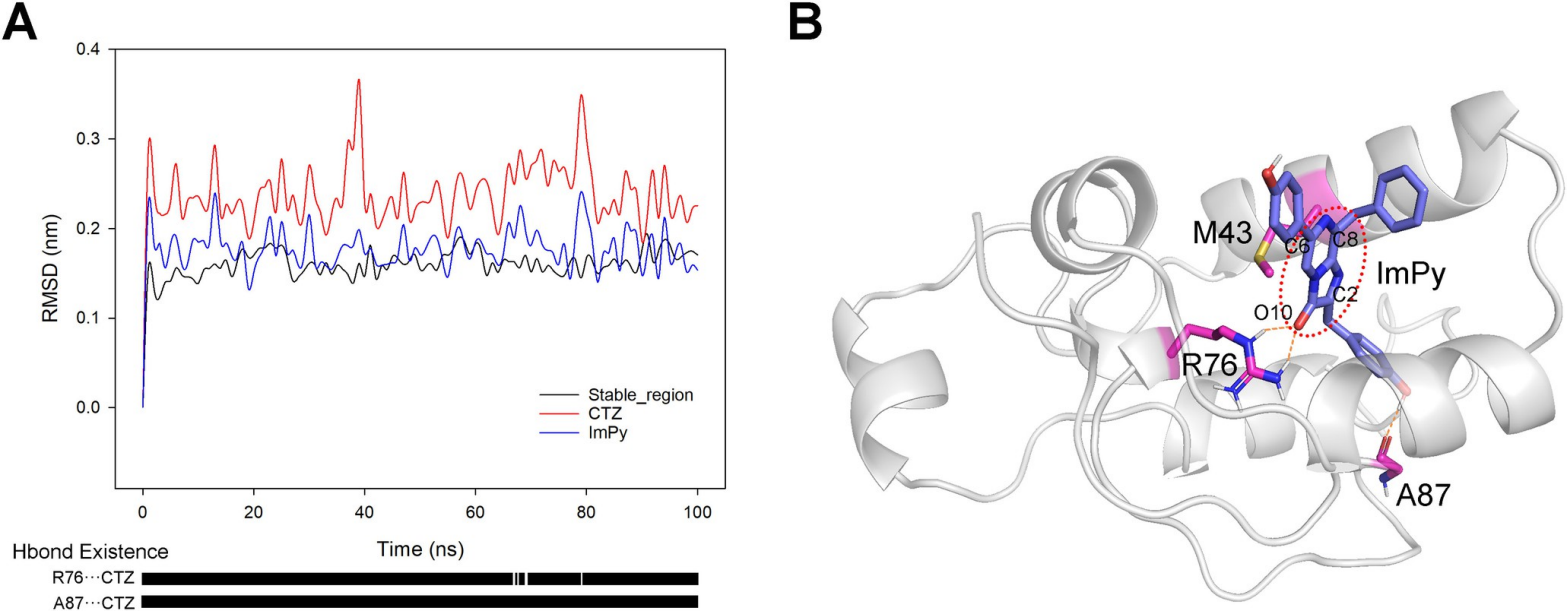
A 100 ns MD simulation and the representative structure of tGLuc-CTZ complex. (A) The upper panel illustrates the time evolution of RMSD of tGLuc’s stable_region, CTZ, and CTZ’s ImPy during the MD simulation. The lower panel shows the hydrogen bond occupancy between R76, A87, and CTZ throughout the simulation. (B) displayed the representative structure of the tGLuc-CTZ complex, with CTZ highlighted in purple and residues M43, R76, and A87 marked in pink.

The representative structure of tGLuc was docked with CTZ using molecular docking, and the optimal docked complex was chosen (see S6 Fig, and the coordinates file was provided in the S1 Supplemental Structures, Docked_complex.pdb) for a 100 ns MD simulation. The RMSD of stable_region kept below 0.2 nm during simulation, demonstrating exceptional stability. Although the RMSD of CTZ exhibited a peak amplitude around 0.35 nm, its reactive group ImPy remained consistently stable at approximately 0.2 nm, indicating that the tGLuc-CTZ complex reached equilibrium (Fig 4A). Hydrogen bond analysis showed that CTZ maintained stable hydrogen bonds with R76 and A87 throughout the simulation (lower panel of Fig 4A). Subsequently, PCA analysis of the 100 ns simulation trajectory identified the lowest-energy complex conformation as the representative structure (Fig 4B). The structure reveals that the phenolic hydroxyl group of C2G forms a stable hydrogen bond with the backbone carbonyl of A87. However, as the backbone carbonyl is not unique to A87 and other docking result suggest the potential for C2G to form hydrogen bond with backbone carbonyl of other amino acid residue (see S6A Fig and S1 Supplemental Structures, Docked_complex_2nd.pdb), the A87 may not irreplaceable for anchoring CTZ within the catalytic cavity. R76, the other residue forming a stable hydrogen bond with CTZ, specifically interacts with the O10 of ImPy through its distinctive side-chain guanidinium group. R76 resides within the highly conserved α5 region (P74–C77, S1 Fig), which is stabilized by the C65/C77 disulfide bond. Previous studies have demonstrated that mutations at either R76 or C77 lead to complete loss of GLuc activity [10,11], identifying this region as a core functional domain and R76 as a key catalytic residue. Additionally, another experimentally validated active residue, M43 [18–21], is located in proximity to the ImPy ring.

**Fig 5:**
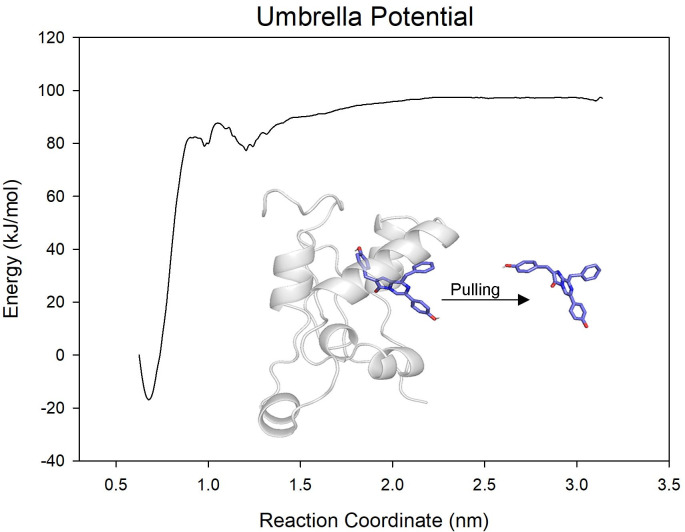
The US analysis on the binding energy of CTZ towards tGLuc.

We then performed US simulations on the CTZ within the representative structure to calculate the binding energy. The result indicated a relatively gradual energy diminish as the centroid distance between CTZ and tGLuc decreased in the initial stage (3.20 nm to 1.20 nm). After a slight fluctuation between 1.20 nm and 0.90 nm, the energy rapidly dropped to a minimum at 0.68 nm. The total binding free energy during this process reached 114 kJ/mol (Fig 5), revealing the strong binding affinity of the tGLuc catalytic cavity for CTZ.

### 3.3. Active_cluster construction and the initial reactant for CTZ oxidation

**Fig 6:**
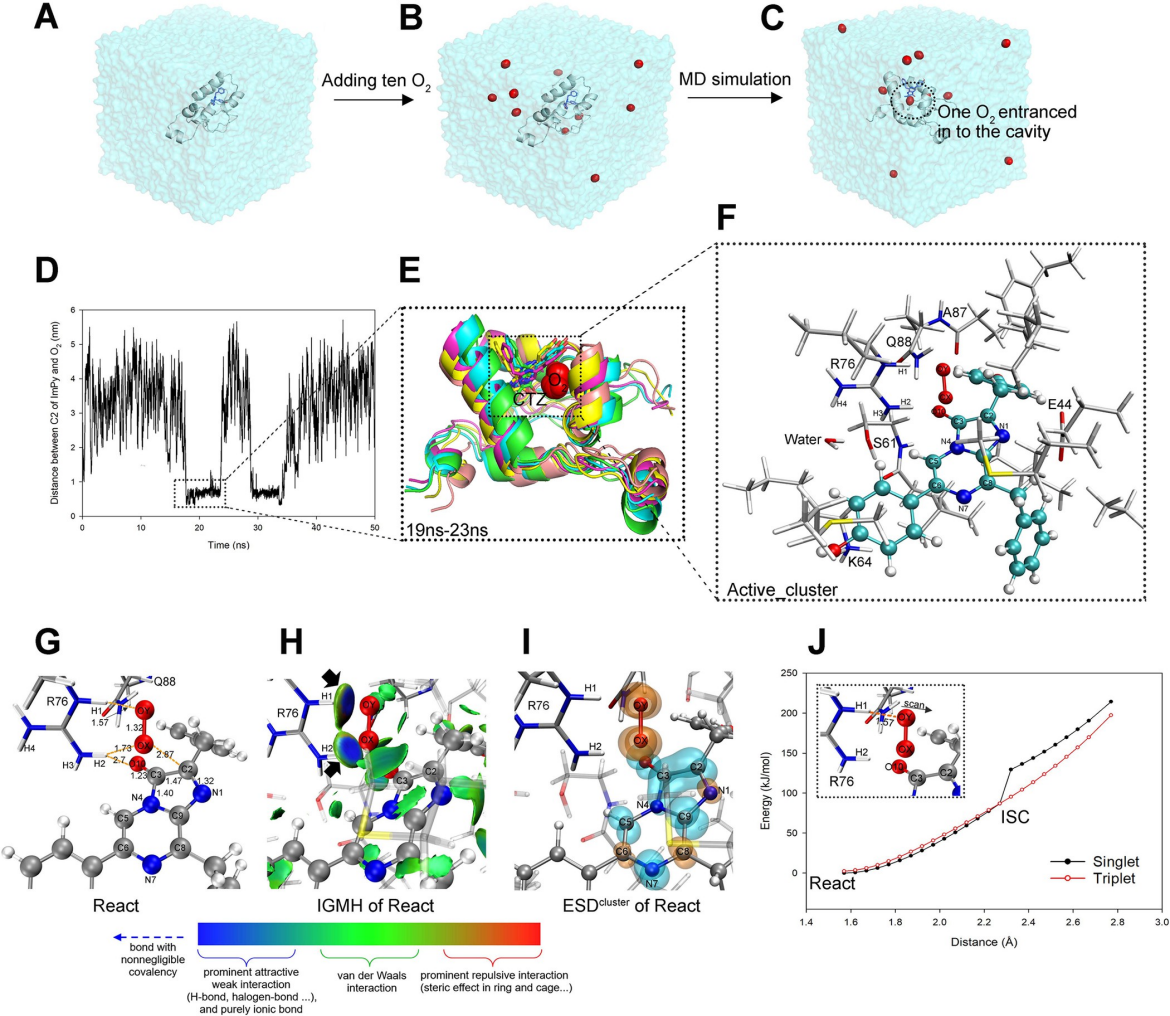
The incorporation of O_2_ into the tGLuc-CTZ complex and the construction of the active_cluster model. (A) displays the representative frame derived from a 100ns MD simulation trajectory of tGLuc-CTZ complex. (B) indicates ten O_2_ were randomly placed around the complex in the frame. Then, a 50ns MD simulation was performed, during which one of the O_2_ entered the tGLuc’s cavity (C). (D) illustrates the distance between the O_2_ and the C2 of ImPy of CTZ. Between 19ns and 23ns, the O_2_ accessed the catalytic cavity and approached ImPy. (E) depicts the conformation of tGLuc-CTZ-O_2_ complex that was superimposed every 1ns during 19ns-23ns. (F) illustrates all atoms of the active_cluster that was constructed by taking the frame with the shortest distance between O_2_ and C2 in the 19ns -23ns trajectory. The active_cluster underwent geometry optimization under the mixed basis set, and (G) shows the optimization result, React, in which only the ImPy, O_2_, two essential residues R76 and Q88 were shown. The distance units here and in the following figures are given in Å. (H) demonstrates the inter-molecular IGMH analysis result. The meaning of isosurface colors in this and subsequent IGMH figures are explained in the bottom color bar. (I) indicates the ESD^cluster^ analysis results of React, where the α and β electrons in this and subsequent ESD figures are marked in transparent orange and cyan, respectively. (J) exhibits the rigid scan results of the distance between O_2_ and the R76’s guanidine group (as shown in the inset), with the ISC point of the singlet and triplet states indicated in the figure. In all geometric structures here and in the following figures, amino acid residues were displayed in stick model, proxy-CTZ was displayed in ball-and-stick model; carbon, nitrogen, hydrogen, and oxygen atoms were colored gray, blue, white, and red, respectively (for reader’s convenience, only carbons of CTZ in (F) were colored in cyan).

To enable the subsequent DFT analysis of the oxidation mechanism of CTZ by tGLuc, it was essential to introduce O_2_ into the tGLuc-CTZ complex obtained in Section 3.2. This was achieved by adding an excess of O_2_ to the environment of tGLuc-CTZ and performing MD simulations to bring one O_2_ enter catalytic cavity and close to CTZ. This approach avoided potential energy conflicts that could arise from manually inserting O_2_ into the catalytic center [12]. A PCA analysis was performed on the 100 ns MD trajectory described in Section 3.2, and the lowest-energy frame of the tGLuc-CTZ complex was selected (Fig 6A). Ten random water molecules in this frame were replaced with O_2_ (Fig 6B), and a 50 ns MD simulation was followed. The analysis of the 50 ns trajectory showed the successful entry of one O_2_ twice (Fig 6C and 6D), at 19 ns- 23 ns and 28 ns- 35 ns, driven by thermal motion into the tGLuc catalytic cavity, close to the initial reaction site (C2 of ImPy, see Fig 6E). Moreover, a water distribution analysis around CTZ showed a very low water content within a 3.5 Å radius of the imidazole (which undergoes chemical changes during ImPy oxidation) of CTZ throughout the 50 ns simulation (S7 Fig). Therefore, all subsequent quantum mechanics calculations were performed in the gas phase.

From the simulated trajectory of the GLuc-CTZ-O_2_ complex, we extracted the frame with the shortest O_2_-C2 distance, and in this frame, the molecules and amino acid residues within a 3.5 Å radius of CTZ were selected to construct the active_cluster (removing redundant waters and amino acids with little relevance to the oxidation of CTZ to speed up the calculation, see Fig 6F). The geometry optimization was performed using the GFN2-xTB (for initial optimization) and DFT methods, resulting in the structure of the initial reactant of the CTZ oxidation in the active_cluster, named React (Fig 6G, the coordinates file was provided in the S1 Supplemental Structures, Active_cluster_React.pdb). The IGMH of React showed that the R76 captured the O_2_ by its guanidine group through strong non-covalent interactions (Fig 6H), with the distances of R76-H1···OY and R76-H2···OX being only 1.57 Å and 1.73 Å, respectively (Fig 6G). React was a biradical singlet with <S^2^> at 1.01, and the ESD^cluster^ indicated that its α electron and β electron were mainly distributed on the O_2_ and ImPy, respectively (Fig 6I). Then we performed a distance scan between R76-H1 and OY (scan range: 1.57 Å -2.77 Å), and an Intersystem Crossing (ISC) from triplet to singlet for the active_cluster was identified at 2.3 Å, indicating that the cavity-entered O_2_ was directly attracted to the guanidine group of R76, and during this process, the natural triplet O_2_ was converted to the biradical singlet state, which had lower system energy (Fig 6J). The occurrence of ISC was verified in the oxidation process of various luciferins, such as D-luciferin catalyzed by firefly luciferase [29] and coelenterazine disulfonate catalyzed by the luciferase in *W*. *scintillans* [30].

### 3.4. The overall oxidation pathway of CTZ within the active_cluster

**Fig 7:**
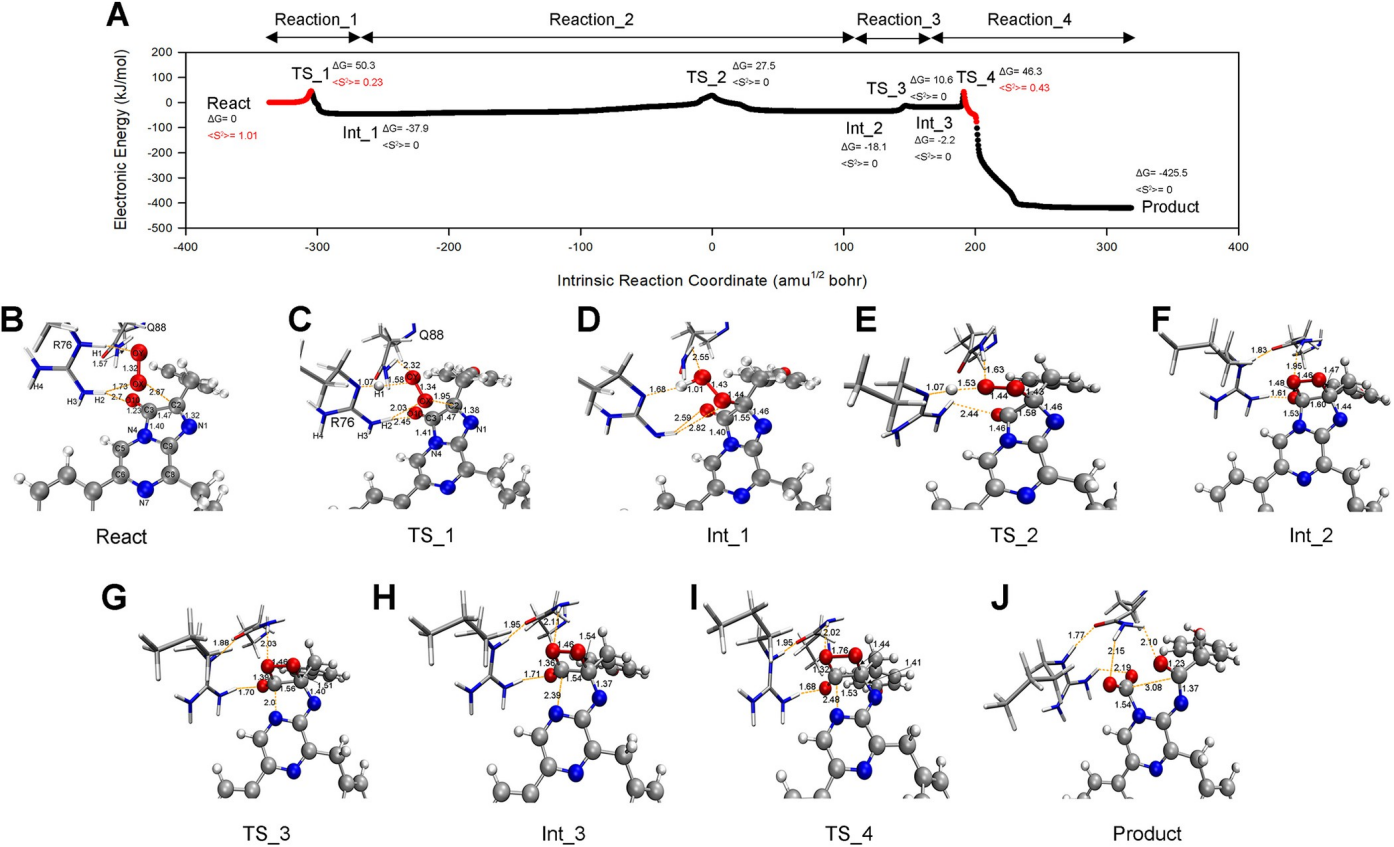
The overall S0 PES for CTZ oxidation in the active_cluster. (A) shows the S0 PES that was constructed under the level of UωB97XD/mix basis set, where the biradical regions were marked in red. The ΔG (unit in kJ/mol) and <S^2^> that indicated on the PES were reevaluated under UωB97XD/6-31+G(d, p) level with the addition of the thermal corrections of 298.15 K and 1 atm as described in the methodology (2.2.3). The geometric structures of R76, Q88, and oxygenated ImPy among the React, all Ints, TSs and the Product are illustrated in (B)-(J).

The S0 PES for CTZ oxidation within the active_cluster was delineated through TS searching and IRC computations (Fig 7A), and thereby four elementary reactions (Reaction_1–4) involving three Ints (Int_1–3), four TSs (TS_1–4), and the final product (Product) were finally identified. The Gibbs energy (ΔG) barriers for the four reactions were: ΔG_TS_1_-ΔG_React_ = 50.3 kJ/mol (Reaction_1), ΔG_TS_2_-ΔG_Int_1_ = 65.4 kJ/mol (Reaction_2), ΔG_TS_3_-ΔG_Int_2_ = 28.7 kJ/mol (Reaction_3), and ΔG_TS_4_-ΔG_Int_3_ = 48.5 kJ/mol (Reaction_4). Clearly, Reaction_2 was the rate-determining step. However, in previous studies on ImPy oxidation excluding surrounding residues [30], Reaction_1 was identified as the rate-determining step, corresponding to O_2_ addition to ImPy to form 2-proxy-ImPy. This process involves both the energy barrier from React to TS_1 in the singlet PES and the energy required for triplet-to-singlet conversion. We computed the oxidation of CTZ alone, from React to Int_2, and found that the ΔG barrier for triplet-to-singlet conversion was 70.9 kJ/mol, while the overall ΔG barrier for the formation of Int_2 was 87.3 kJ/mol (see S8 Fig). This aligns with the known fact that CTZ exposed to air does not readily convert to proxy-CTZ. As previously mentioned, the O_2_ attracted by R76 has spontaneously formed a singlet state via ISC. Therefore, the oxidation of ImPy within the active_cluster only requires overcoming a barrier on the singlet PES, which is 65.4 kJ/mol, significantly reducing the barrier. This is likely the key mechanism through which tGLuc catalyzes and accelerates the reaction.

The detailed mechanism of CTZ oxidation and actions of R76 and another highly conserved polar residue Q88 (S1 Fig), which were close to R76 and ImPy and played catalytic roles, are explained below.

#### 3.4.1. The binding of O_2_ towards ImPy (Reaction_1)

**Fig 8:**
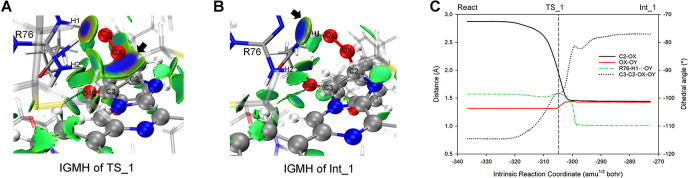
IGMH and geometry analysis along the S0 PES of Reaction_1. (A) and (B) display the inter-molecular IGMH isosurfaces of TS_1 and Int_1, respectively. (C) shows the crucial geometric parameters along the coordinate of Reaction_1.

We manually directed the oxygen atom (OX) of the O_2_ molecule towards the ImPy within the React (Fig 7B), thus identified the TS_1 (Figs 7C and 8A) for Reaction_1. Vibrational analysis verified the imaginary frequency of TS_1 at -397.65 cm^-1^, indicating the approach of the OX towards ImPy’s C2 carbon. Based on TS_1, IRC calculations revealed that Reaction_1 connected the React and Int_1 (Figs 7D and 8B), characterized by the OX detaching from R76-H2 and migrating towards C2 of ImPy. C2-OX decreased from 2.87 Å in the React to 1.98 Å in TS_1 (Fig 8C), accompanied by a decline in the <S^2^> value from 1.01 to 0.23 (Fig 7A). This decrease in <S^2^> is attributed to the proximity of α electrons on the O_2_ to β electrons on ImPy, increasing the attraction between the OX and C2, as demonstrated by the IGMH of TS_1 (Fig 8A). Following this, Int_1, the product of this elementary reaction, displayed a covalent bond between the OX and C2, with a bond length of 1.44 Å (Fig 7D), and featured both the spin annihilation (Fig 7A) and the extension of the OX-OY bond from 1.34 Å in TS_1 to 1.43 Å (Fig 8C), giving rise to a 2-proxy-CTZ. This intermediate subsequently evolved into a stable 2-hydroproxy-CTZ as OY acquired a proton released from the guanidine group of R76, evidenced by the contraction of the R76-H1···OY distance to 1.01 Å (Fig 8C). The deprotonated R76 guanidine manifested a potent attraction towards the hydroxyl group (Fig 8B). Throughout the Reaction_1, the dihedral angle encompassing C3-C2-OX-OY escalated from -114.5° in the React to -77° in Int_1 (Fig 8C).

We computed the triplet PES of Reaction_1 and observed that the singlet and triplet PES, initially close at React due to R76 attracting O_2_ (Fig 6J), diverged near TS_1, with the singlet energy subsequently dropping significantly below the triplet (S9 Fig). Additionally, we constructed an active_cluster with the R76A mutation and, through IRC calculations, confirmed that in the absence of R76, the ΔG gap between the singlet and triplet Reacts reached 46 kJ/mol, with the oxygenation ΔG barrier rising to 98.8 kJ/mol, significantly higher than that of induced by R76 (S10 Fig). This highlights R76 as the key to the active cluster’s catalytic function.

#### 3.4.2. The formation of Dioxetanone Intermediate (Reaction_2 and Reaction_3)

**Fig 9:**
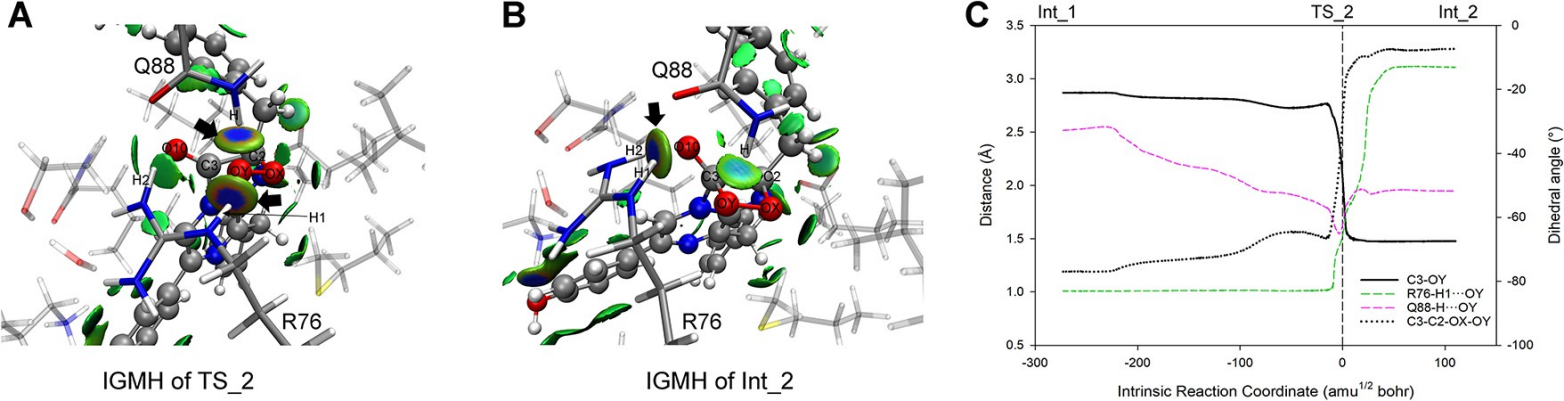
IGMH and geometry analysis along the S0 PES of Reaction_2. (A) and (B) display the inter-molecular IGMH isosurfaces of TS_2 and Int_2, respectively. (C) shows the crucial geometric parameters along the coordinate of Reaction_2.

We adjusted the dihedral angle C3-C2-OX-OY of Int_1 and identified TS_2 (Figs 7E and 9A) for Reaction_2 at -29.85°. Vibrational analysis confirmed TS_2’s imaginary frequency at -117.80 cm^-1^, and the IRC calculation indicated that Reaction_2 bridged the Int_1 and Int_2 (Figs 7F and 9B). In the course of Reaction_2, the dihedral angle C3-C2-OX-OY rotating counterclockwise around the C2-OX axis, transitioning from -77° in Int_1 to -7° in Int_2, where the C3-OY contracted to 1.48 Å (Fig 9C), resulting in the formation of a four-membered ring (dioxetanone, see Fig 7F). The rotation of C3-C2-OX-OY was most significant near TS_2, within the range of -15 to 15 amu^1/2^ bohr, where R76-H1 disengaged from OY (Fig 9C, see R76-H1···OY) and reassociated with the guanidine group of R76. At TS_2, the deprotonated OY, exhibiting high electronegativity, attracted the adjacent amide hydrogen of Q88 (see Q88-H···OY in Fig 9C) and R76-H1 for charge stabilizing (Fig 9A) until OY nucleophilically attacked C3. Following the closure of the C2-C3-OY-OX framework, the surplus electrons from OY were transferred to O10 (S11 Fig), thus the resultant Int_2 displayed diminished interactions at R76-H1···OY and Q88-H···OY, and an intensified interaction at R76-H2···O10 compared to TS_2 (Fig 9A and 9B).

**Fig 10:**
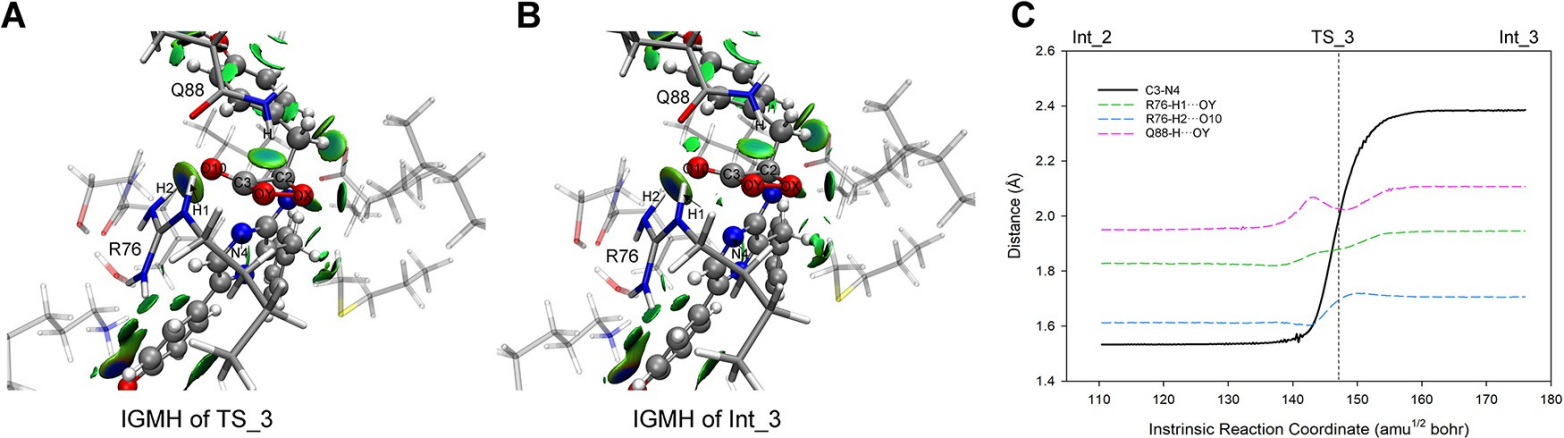
IGMH and geometry analysis along the S0 PES of Reaction_3. (A) and (B) display the inter-molecular IGMH isosurfaces of TS_3 and Int_3, respectively. (C) shows the crucial geometric parameters along the coordinate of Reaction_3.

Subsequently, we varied the C3-N4 bond length in Int_2 and identified the TS_3 (C3-N4 = 2.00 Å, see Figs 7G and 10A) for Reaction_3. Vibration analysis confirmed a TS_3 imaginary frequency at -128.29 cm^-1^, associated with the elongation of the C3-N4 bond. IRC computation revealed the Reaction_3 bridged the Int_2 and the Int_3 (Figs 7H and 10B), and indicated a gradual increase in the C3-N4 bond length from 1.53 Å to 2.39 Å (Fig 10C), leading to the bond’s cleavage and the emergence of an anionic dioxetanone Intermediate (dioxetanone^-^) in Int_3. Throughout Reaction 3, despite slight distance increases in the R76-H1···OY, Q88-H···OY, and R76-H2···O10 (Fig 10C), the IGMH suggested negligible alterations in the non-covalent interactions among these atom pair (Fig 10A and 10B).

#### 3.4.3 CIEEL reaction on dioxetanone^-^ (Reaction_4)

**Fig 11:**
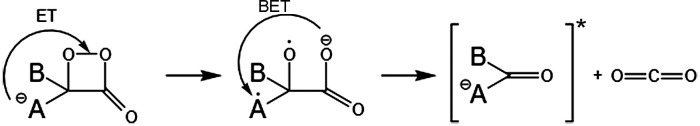
Excited-state formation based on intra-molecular ET/BET from a dioxetanone^-^.

Recent ImPy oxidation studies indicate that dioxetanone can produce excited-state products via the CIEEL reaction [5]. The mechanism involves a linkage of the carbon in dioxetanone to a negative charge moiety, acting as an electron donor. This triggers Electron Transfer (ET), resulting in the breaking of the peroxide bond and the creation of a biradical anion state. Then, Back Electron Transfer (BET) takes place, causing the cleavage of the carbon-carbon bond. The aforementioned process is adept at generating singlet-state excited ketone products, which emit photons effectively during the de-excitation process [29,62] (Fig 11).

**Fig 12:**
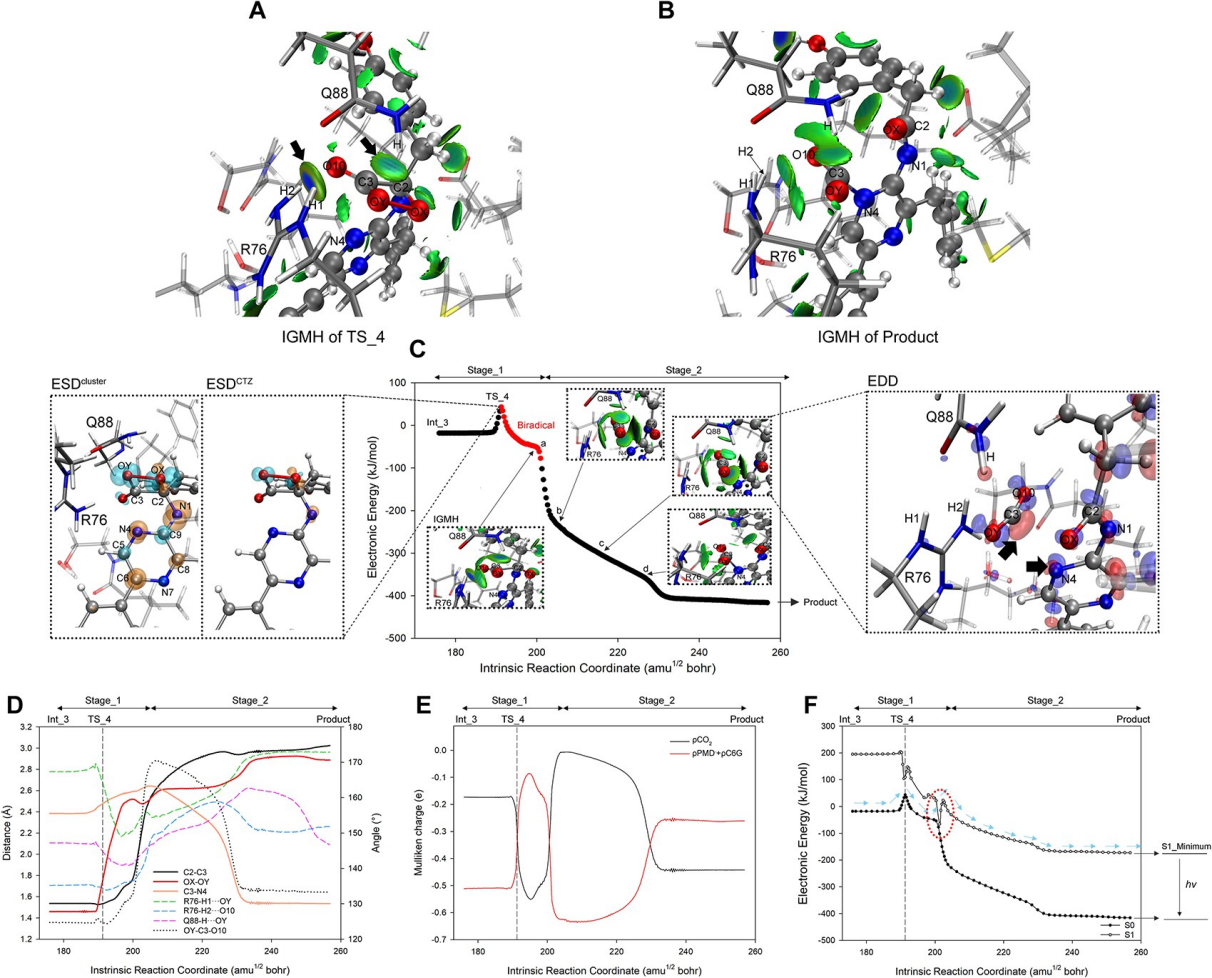
Geometry, IGMH, ET/BET, and S1/S0 PES analysis along the coordinate of Reaction_4. (A) and (B) display the inter-molecular IGMH isosurfaces in TS_4 and Product, respectively. (C) illustrates the S0 PES along the reaction coordinate, with the biradical region marked in red. The inset figures show the inter-molecular IGMH isosurfaces at points (a), (b), (c), and (d) along the S0 PES: points (a) and (d) present isosurfaces between proxy-CTZ and surrounding residues due to CO_2_ binding to PMD^-^, while points (b) and (c), where CO_2_ detaches from PMD^-^, present isosurfaces among PMD^-^, CO_2_, and surrounding residues. The analysis results of ESD^cluster^ and ESD^CTZ^ at TS_4, the onset of the biradical region, are depicted in the left panel. The EDD analysis at the point (b) is depicted in the right panel, with regions of increased electron density in transparent blue and decreased electron density in transparent red. (D) indicates the crucial geometric parameters of the S0 PES along the reaction coordinate. (E) indicates the ET/BET between the CO_2_ moiety and PMD^-^ + C6G (C6G is included here due to its participation in π-bond-based electron transfer with PMD^-^) moiety. (F) demonstrates the S1 PES generated from the geometries of scatter points on the S0 PES, with the closest between S0 and S1 PES denoted by a red dotted circle.

We adjusted the OX-OY bond length in Int_3 and identified the TS_4 (Figs 7I and 12A) for Reaction_4. Vibration analysis confirmed the imaginary frequency of TS_4 as -1455.41 cm^-1^, corresponding to the stretching of the OX-OY bond. The IRC calculation disclosed the Reaction_4 linked Int_3 and Product (Figs 7J and 12B), comprising two stages (Fig 12C). The stage_1 (176–204 amu^1/2^ bohr) involved dioxetanone^-^ dissociation, during which the covalent bonds OX-OY and C2-C3 in dioxetanone^-^ ruptured consecutively (OX-OY lengthened swiftly from 1.46 Å to 2.50 Å, succeeded by a lengthening in C2-C3 from 1.54 Å to 1.80 Å, as illustrated in Fig 12D), resulting in the detachment of OY-C3-O10 (CO_2_) moiety from the residual anion PMD (PMD^-^) moiety. During this period, the system manifested biradical characteristics (Fig 7A and 12C, 191–204 amu^1/2^ bohr). In TS_4 (at 191 amu^1/2^ bohr), the onset of the biradical stage, radical electrons were allocated on the oxygenated-ImPy^-^ (Fig 12C, left panel, ESD^cluster^), followed by a charge decrease in CO_2_ moiety and a charge increase in PMD^-^ moiety during 191–195 amu^1/2^ bohr, implying an electron transfer from PMD^-^ moiety to CO_2_ moiety, indicative of an ET process. In the interval of 195–204 amu^1/2^ bohr, there was a charge increase in CO_2_ moiety and a charge decrease in PMD^-^ moiety, signifying electron transfer from CO_2_ moiety back to PMD^-^ moiety, constituting a BET process (Fig 12E). In the above electron exchange between CO_2_ moiety and PMD^-^ moiety, the S1 PES approached the S0 PES at 201 amu^1/2^ bohr, with an energy gap merely of 36 kJ/mol. Thus, the system could cross this vicinity from S0 to S1 (Fig 12F). We analyzed the charge and free radical electron distribution on the dioxetanone ring and other components of CTZ and found the above process strictly conformed to the CIEEL theory (see S13 Fig) and was consistent with various reported ImPy-based bioluminescence mechanisms [30,31,63]. Additionally, we observed R76 and Q88 in the active_cluster provided strong non-covalent interactions with the dioxetanone^-^ in TS_4 (Fig 12A), and R76-H1···OY, Q88-H···OY and R76-H2···O10 reached their minimum distance during the electron exchange (Fig 12D, 191–204 amu^1/2^ bohr). To examine the roles of R76 and Q88 in CIEEL, we calculated the ESD^CTZ^ at TS_4 and compared it with the ESD^cluster^, the result revealed R76 and Q88 significantly enhanced the ESD of dioxetanone^-^ (Figs 12C left panel, and S12A), promoting the ET/BET and facilitating the efficiency of excited ketone production according to the CIEEL theory.

Upon completing the CIEEL process, the system reverted to a closed-shell state, commencing the stage_2: the reconstitution of the C3-N4 bond (Fig 12C, 204 amu^1/2^ bohr—the end of Reaction_4). Following the complete cleavage of OX-OY and C2-C3 bonds, the OY-C3-O10 angle expanded to its zenith of 170° at 205 amu^1/2^ bohr, indicating the CO_2_ moiety finally detaching from PMD^-^ moiety (Fig 12D). Typically, in ImPy oxidation experiments conducted outside of enzymatic settings, the liberated CO_2_ diffused into the environment. Contrastingly, IGMH analysis revealed that within the active_cluster, the separated CO_2_ was enclosed by non-covalent forces emanating from the PMD^-^, R76, and Q88, impeding its egress from the catalytic chamber (Fig 12C inset a-b). In tandem, the affinity between the N4 nitrogen of the PMD^-^ and the C3 carbon of CO_2_ intensified post-205 amu^1/2^ bohr, drawing the CO_2_ closer to the PMD^-^ moiety, culminating in the formation of the C3-N4 bond (1.5 Å) at 233 amu^1/2^ bohr (Fig 12C inset c-d), concurrent with the reduction of the OY-C3-O10 angle to 133° (Fig 12D). Charge analysis indicated that the electrostatic charge of separated CO_2_ at 205 amu^1/2^ bohr neared neutrality, then diminished progressively. This was mirrored by an incremental charge accumulation on the PMD^-^ moiety. The synchronous charge fluctuations suggested an incremental negative charge transfer from the PMD^-^ to the CO_2_, underscoring the predominance of electrostatic attraction in the C3-N4 reconstitution (Fig 12E). It should be clarified that the charge crossover at 233 amu^1/2^ bohr in Fig 12E represents mere charge transfer from PMD^-^ to CO_2_, with no involvement of free radical electrons (Fig 12C) or PES crossings (Fig 12F), thus unrelated to the CIEEL reaction (which, in fact, finished at Stage_1). An EDD examination of the intermediary structure (Fig 12C, inset c, at 215 amu^1/2^ bohr) during the C3-N4 reconstitution phase revealed that the N4 to C3 attraction stemmed from the influence of R76 and Q88, which localized the CO_2_ electrons at the termini of OY and O10, rendering the central C3 electron density deficient and electrophilic. Concurrently, the electron transfer from N1 of the PMD^-^ moiety to N4 engendered a robust electrostatic pull between N4 and C3 (Fig 12C, right panel), ultimately leading to their union via nucleophilic addition.

**Fig 13:**
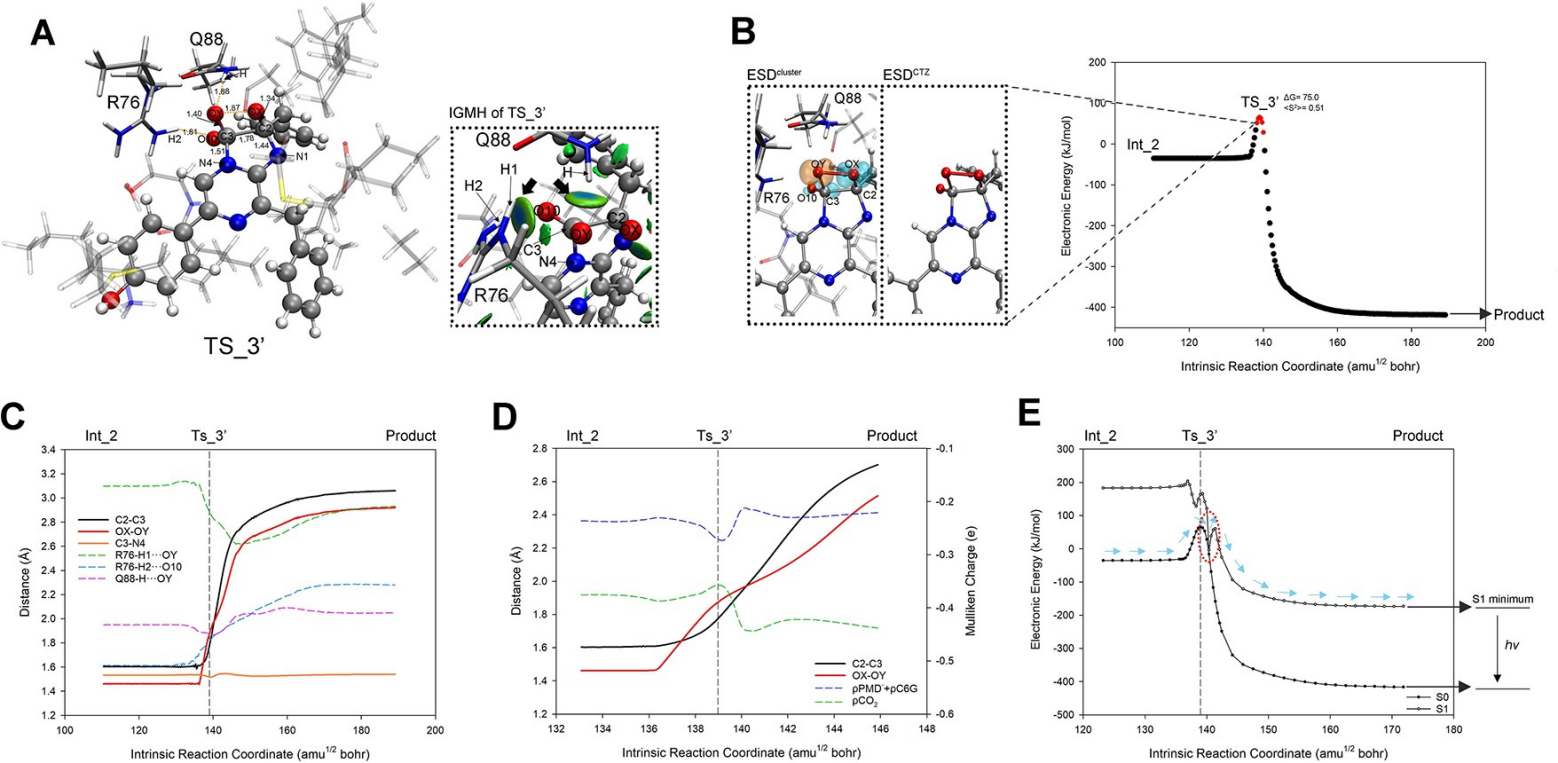
Geometry, IGMH, ET/BET, and S1/S0 PES analysis along the coordinate of Reaction_3’. (A) shows the geometry and IGMH isosurfaces between proxy-CTZ and surrounding residues in TS_3’. (B) displays the S0 PES along the reaction coordinate, with the biradical region marked in red. The analysis results of ESD^cluster^ and ESD^CTZ^ at the beginning of the biradical region (138 amu^1/2^ bohr) are depicted in the left panel. (C) indicates the crucial geometric parameters of the S0 PES along the reaction coordinate. (D) illustrates the ET/BET between the CO_2_ moiety and PMD^-^ + C6G moiety along with the bond lengths of C2-C3 and OX-OY. (E) demonstrates the S1 PES generated from the geometries of selected scatter points on the S0 PES, with the closest between S0 and S1 PES denoted by a red dotted circle.

The C3-N4 bond that ruptured in Reaction_3 reconnected within the active_cluster in Reaction_4, prompting us to explore the possibility of CIEEL reaction occurring directly from Int_2 to the Product. Thus, by varying the OX-OY distance of Int_2, we located TS_3’ (Fig 13A) where CIEEL reaction occurred under the condition of C3-N4 bonding. Vibration analysis confirmed TS_3’ with an imaginary frequency of -751.96 cm^-1^, corresponding to the nearly equifrequency elongation of OX-OY and C2-C3 bonds. IRC computation of TS_3’ yielded the S0 PES of Reaction_3’ (Fig 13B), bridging Int_2 and the Product with C3-N4 maintaining a bond distance around 1.54 Å (Fig 13C). Although the stretching of OX-OY and C2-C3 was sequential within Reaction_3’, both initiated almost simultaneously (Fig 13C), resulting in a biradical state within a small range around TS_3’ (Fig 13B, 138–140 amu^1/2^ bohr). The ESD^cluster^ analysis at the starting point of the biradical region (138 amu^1/2^ bohr) revealed the radical electrons were initially distributed on the dioxetanone and O10 (Fig 13B, left panel, ESD^cluster^), and from which, the charge of PMD^-^ moiety decreased while that of CO_2_ moiety increased during the interval of 138–139 amu^1/2^ bohr, signifying the ET, where electron transfer from CO_2_ moiety to PMD^-^ moiety. Then, within the interval of 139–140 amu^1/2^ bohr, PMD^-^ moiety charge increased while CO_2_ moiety charge decreased, signifying the BET, where electrons returned from PMD^-^ moiety to CO_2_ moiety (Fig 13D). In contrast to the scenario of Reaction_4, the electron exchange sequence was completely reversed, with a significantly lower charge transfer amount. The above characteristics indicated the Reaction_3’ resembled the CIEEL reaction of a neutral dioxetanone (N1 bonded with an external proton) [29], which was reported using an entropic trap to bring the S0 PES and S1 PES into proximity [29,31]. Indeed, through TD-DFT analysis of Reaction_3’, we identified the entropic trap at 140 amu^1/2^ bohr with a mere 13 kJ/mol energy gap between the PES of S0 and S1 (Fig 13E). This energy gap was much smaller than that of Reaction_4, leading to the conclusion that oxygenated CTZ could generate excited-state products through Reaction_3’ easier, however, the Gibbs energy barrier of Reaction_3’ calculated by ΔG_TS_3’_-ΔG_Int_2_ was 93.1 kJ/mol, almost two times higher than that of Reaction_4, indicating the Reaction_3’ was unlikely to occur in nature. In addition, R76 and Q88 that were crucial for Reaction_4, played similar significant roles for Reaction_3’, because: (1) R76-H1···OY, Q88-H···OY and R76-H2···O10 reached their minimum distance approaching/within the electron exchange stage (Fig 13C), and pronounced attractive interactions between R76, Q88 and dioxetanone were observed through IGMH analysis on TS_3’ (Fig 13A, right panel); and (2) at the onset of biradical stage (138 amu^1/2^ bohr), the ESD^cluster^ was much higher than the ESD^CTZ^ on the oxygenated-ImPy (Fig 13B left panel and S12B) indicating the ET/BET of CIEEL was enhanced in the active_cluster, same as that of Reaction_4.

**Fig 14:**
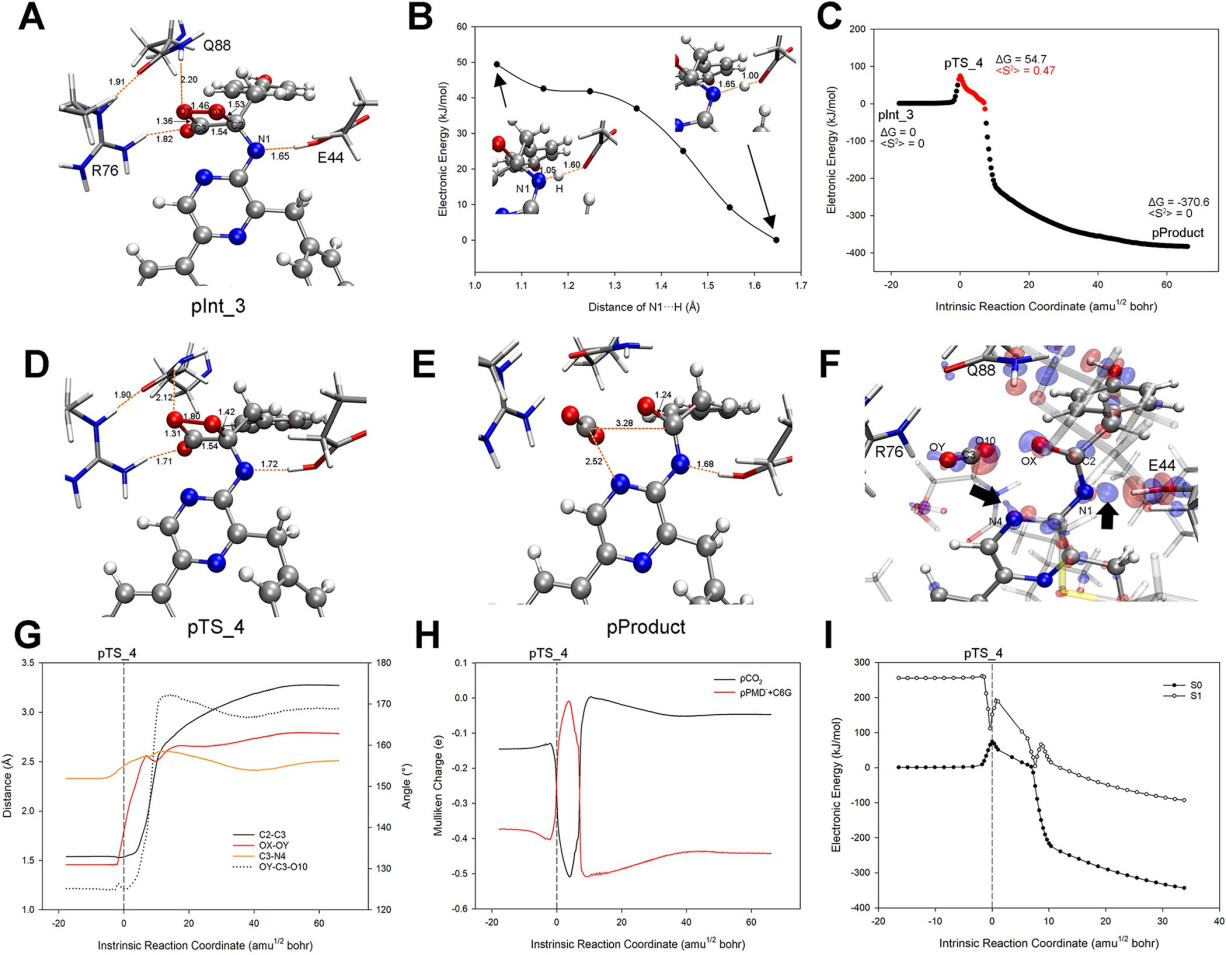
Geometry, ET/BET, and S1/S0 PES analysis along the coordinate of CIEEL reaction that begin with pInt_3. (A) shows the R76, Q88, and oxygenated ImPy in pInt_3; (B) displays the rigid scan of the distance between N1 and the proton in pInt_3 (scanned from 1.05 to 1.65 Å); (C) displays the S0 PES along the reaction coordinate, with the biradical region marked in red; (D) and (E) respectively shows the R76, Q88, and oxygenated ImPy in pTS_4 and pProduct; (F) displays the EDD analysis at the pProduct; (G) indicates the crucial geometric parameters of the S0 PES along the reaction coordinate. (H) indicates the ET/BET between the CO_2_ moiety and PMD^-^ + C6G moiety. (F) demonstrates the S1 PES generated from the geometries of scatter points on the S0 PES.

According to previous studies, the CIEEL reaction based on ImPy oxidation follows two modes: dioxetanone^-^ mode and neutral dioxetanone mode. The former corresponds to the mechanism of Reaction_4, where N1 of ImPy participates in the CIEEL reaction as an anion (Int_3). The latter occurs when N1 binds a proton, leading to a neutral dioxetanone CIEEL reaction, with a significantly different PES from that of Reaction_4 [29–31]. Therefore, we protonated N1 in Int_3 to construct protonated Int_3 (pInt_3) and attempted to calculate the PES of neutral dioxetanone based on it. However, after geometric optimization, we found that the proton initially bound to N1 of pInt_3 was transferred to the carboxyl group of E44 (the optimized structure showed ImPy-N1···H···OOC-E44 nearly aligned, see Fig 14A). Consequently, we scanned the distance between N1 and the proton in the geometric optimized pInt_3. The results revealed a gradual decrease in system energy as the distance between N1 and the proton increased (Fig 14B), until the proton was bonded with the carboxyl group of E44 (H···OOC-E44 = 1.0 Å). The reported optimal pH for tGLuc catalysis is neutral [28], thereby the deprotonated carboxyl group of E44 should maintain the N1 to be deprotonated, and the dioxetanone involved in electron exchange tends to remain in an anion state, consistent with Yue’s findings [63]. Subsequently, using pInt_3, we identified the TS (pTS_4) and Product (pProduct) of the protonated CIEEL reaction, establishing the complete reaction pathway (Fig 14C). The overall PES resembled that of Reaction_4 but had a slightly higher ΔG reaction barrier of 54.7 kJ/mol. The geometry of pTS_4 was similar to TS_4 (Fig 14D), but the pProduct showed no bond formation between C3 and N4, with complete dissociation of CO_2_ and PMD^-^, differing significantly from the Product (Fig 14E). EDD analysis indicated that protonation of E44, which accumulated PMD^-^ electrons around N1, hindered N4 from acquiring sufficient electrons, preventing electrostatic attraction between N4 and C3 (Fig 14F). Further structural and electronic analysis confirmed that this reaction is analogous to Reaction_4: the sequential cleavage of OX-OY and C2-C3 occurred in a 12 amu^1/2^ bohr range (-2-10 amu^1/2^ bohr, see Fig 14G), and the same electron exchange sequence as Reaction_4 between the CO_2_ and PMD^-^ moieties took place (Fig 14H). During the electron exchange, a radical electron appeared (Fig 14C), and at 7 amu^1/2^ bohr, a close proximity of S0 and S1 PES responsible for chemiluminescence was observed (Fig 14I). Since N4 lacked electrons to attract C3, after the initial separation of CO_2_ and PMD^-^ at 10 amu^1/2^ bohr, electron exchange did not occur as in Reaction_4 (Fig 14H), and C3-N4 was ultimately unbonded. The OY-C3-O10 angle stabilized at 170° (Fig 14G), indicating that C3 retained its sp2 hybridization. The PES of this CIEEL reaction differs significantly from the reported neutral dioxetanone PES, suggesting that in the presence of E44, CTZ’s CIEEL reaction consistently proceeds via the dioxetanone^-^ mode.

Additionally, we conducted PES calculations for Reaction_4 in a non-catalytic environment under anionic and protonated conditions. The results showed that the S0 reaction barrier of dioxetanone in its protonated state reaches 95.5 kJ/mol (S14 Fig), nearly twice that of the anionic state. Such a high barrier would result in an extremely slow CIEEL process at room temperature. However, it has been confirmed through real experiments that the tGLuc-catalyzed CTZ reaction proceeds rapidly [28]. Therefore, we propose that dioxetanone undergoes the CIEEL reaction in its anionic state within tGLuc. Protonation at N1 may occur in coelenteramide, as reported by Chen et al. [64].

#### 3.4.4 The hydrogen bond net around oxygenated-CTZ in active_cluster

**Fig 15:**
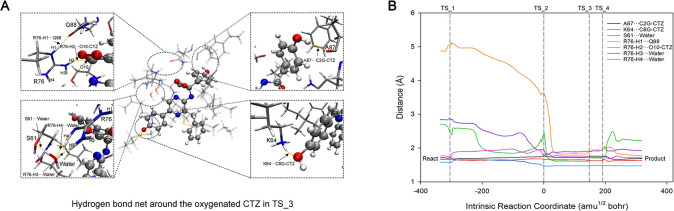
Illustration of hydrogen bond network around the oxygenated CTZ in TS_3 (A) and length variations of these hydrogen bonds along the coordinate of Reaction_1–4 (B).

The active_cluster includes additional amino acids beyond R76 and Q88 that interact with CTZ. A87, as evidenced by MD simulations, forms a persistent hydrogen bond with the phenolic hydroxyl group of C2G (see Fig 4), maintaining this interaction throughout the DFT reaction pathway (Fig 15, A87···C2G-CTZ under 1.8 Å). K64’s side chain, while potentially forming a hydrogen bond with C6G’s phenolic hydroxyl group (Fig 15, K64···C6G-CTZ), exhibits significant flexibility and is exposed to water in MD simulations (S7 Fig), suggesting challenges in maintaining this bond in a fluid, high-dielectric environment. S61, not directly interacting with CTZ in DFT studies, forms a stable hydrogen bond with a water molecule (Fig 15, S61·Water below 1.7 Å), which then connects with R76-H4 and R76-H3 after the dioxetanone formation in Reaction_2 (beyond 15 amu^1/2^ bohr). Additionally, R76-H1 engages in hydrogen bonding with Q88’s carbonyl (Fig 15, R76-H1···Q88), contributing to a hydrogen bond network involving S61-Water-R76-Q88. This is corroborated by previous Heteronuclear Single Quantum Coherence (HSQC) studies using NMR in deuterium oxide, which identified S61 within a hydrogen bonding network [14]. And Further, Although mutational effects on S61 remain unexplored for GLuc, its analogous position in picALuc, a CTZ luciferase with a sequence homologous to GLuc (S1 Fig), has been proved critical for enzymatic function through mutagenesis [65]. The oxidized CTZ is stabilized near this S61-Water-R76-Q88 network through a hydrogen bond from R76-H2 to ImPy’s O10, ensuring the catalytic residues R76 and Q88 remain close to the dioxetanone, facilitating the CIEEL reaction.

## 4 Conclusion

**Fig 16:**
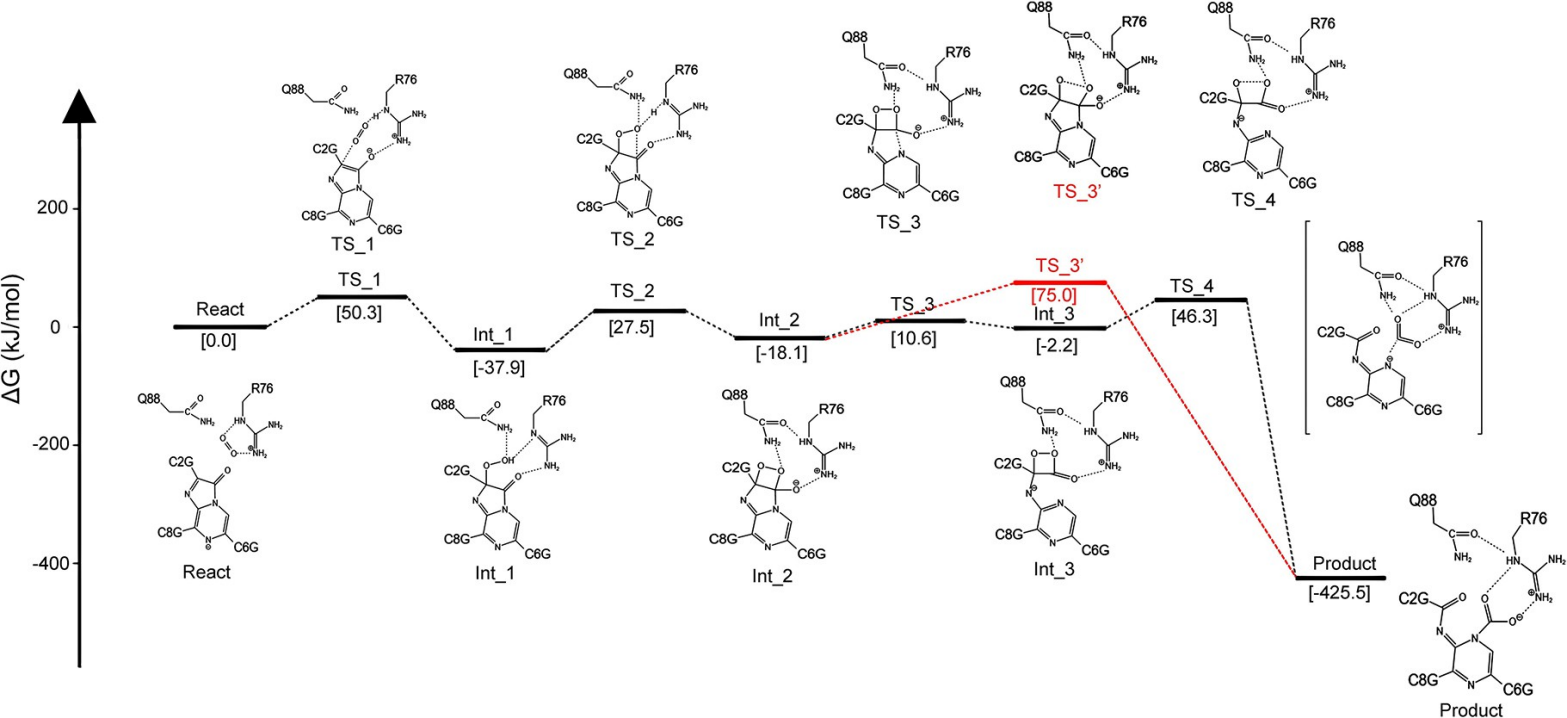
Schematic depiction of the structure and energy variations throughout the CTZ oxidation process within the active_cluster.

Despite the elucidation of GLuc’s structure, its catalytic mechanism remains elusive, as both the NMR and AF2 structures indicate that GLuc is a single-domain enzyme [14,16], yet the fragment splitting assay [7], hill plot analysis [8], strong luminescence intensity of tGLuc [28], and the widespread protein-fragment complementation assay (PCA) based on GLuc [66] suggest that the enzyme has two relatively independent folding units [9]. Here, we truncated the C-terminus of the AF2 structure and found that the remaining tGLuc fragment retained its structure during MD simulation. The central cavity region in the AF2 structure [16] maintained its conformation and formed a stable open-mouthed cavity in tGLuc. The cavity displayed a strong binding affinity for CTZ as revealed by Docking and US simulation analysis, and a DFT calculation using cluster model further confirmed its catalytic activity towards CTZ oxidation. R76, a vital functional residue, attracted a triplet O_2_ that entered the cavity and resulted in a singlet biradical state, that largely decreased the ΔG energy barrier for its addition to ImPy, thereby facilitating the formation of 2-hydroproxy-CTZ. Subsequently, the 2-hydroproxy-CTZ cyclized to a dioxetanone^-^, with the surrounding E44 ensuring that dioxetanone proceeds as an anion in the subsequent CIEEL reaction. During the CIEEL process, the OX-OY and C2-C3 bonds sequentially cleave, accompanied by the generation of free radicals, the ET/BET between CO_2_ moiety and the PMD^-^ moiety, and the proximity between S0 and S1 PESs, resulting in luminescence and the separation of CO_2_ and PMD^-^. The C3-N4 bond can be maintained during CIEEL, but this leads to a higher reaction barrier, thus the CIEEL should preferably occur under bond cleavage condition. The rate-limiting step of the whole ImPy oxidation was Reaction_2, corresponding to the formation of dioxetanone^-^ from the 2-proxy-ImPy. Additionally, R76 and Q88, through an S61-mediated hydrogen bond network, were stabilized near dioxetanone throughout the reaction, exerting their influence by stabilizing reaction TSs and Ints, and enhancing free radical electrons in CIEEL, thereby improving reaction efficiency. Upon completion of CIEEL, the central carbon atom of the dissociated CO_2_ exhibited low electron density due to the induction by R76 and Q88. Consequently, CO_2_ electrophilically added to the N4 atom, ensuring the consistency of the final product regardless of the occurrence of C3-N4 cleavage during the reaction (Fig 16).

In summary, our study identifies a key catalytic residue R76 in tGLuc, along with two auxiliary catalytic residues E44 and Q88. It is important to clarify that due to the refolding observed in the MD simulation, the conformation of tGLuc differs to some extent from the original conformation. Therefore, the functions of E44 and Q88 should be considered specific to tGLuc, aside from the already established key catalytic residue R76 in full-length GLuc. Notably, the central catalytic cavity of full-length GLuc also contains another key catalytic residue R147. The exact mechanism by which the two arginines catalyze CTZ within the same cavity, and whether the auxiliary residues E44 (in fact, the E44A mutation was proven to reduce the luminescence intensity of full-length GLuc to 4% [11]) and Q88 are involved, requires further investigation.

Finally, we emphasize that the CTZ oxidation mechanism elucidated in this study is specific to tGLuc. Given the diversity of CTZ luciferases, some of which lack arginine residues in their catalytic cavities, it is likely that these enzymes utilize distinct catalytic mechanisms.

## Supporting information

S1 Fig Sequence alignment of GLuc and a phylogenetic tree topology of GLuc and selected 15 similar luciferases constructed by maximum likelihood using MEGA11. (DOCX)

S2 Fig The mixed basis set for the active_cluster. (DOCX)

S3 Fig Ramachandran plot of the representative structure of tGLuc and the time evolution of tGLuc and its stable_region’s radius of gyration and solvent-accessible surface area during 500ns MD simulation. (DOCX)

S4 Fig Three independent 500 ns MD simulations starting from the AF2 tGLuc structure with randomized initial velocities. (DOCX)

S5 Fig RMSF of the 200 ns MD simulation trajectory of full-length GLuc. (DOCX)

S6 Fig Molecular docking of tGLuc with CTZ using Autodock4. (DOCX)

S7 Fig The number of water molecules within a radius of 3.5 Å around the Imidazole, Pyridine, C2G, C6G and C8G of CTZ. (DOCX)

S8 Fig Singlet/triplet PES of the isolated CTZ oxidation begin with React to the formation of Int_2 dioxetanone^-^. (DOCX)

S9 Fig Singlet/triplet PES of the Reaction_1. (DOCX)

S10 Fig Singlet/triplet PES of Reaction_1 within alanine substitution on the R76. (DOCX)

S11 Fig The changes of Mulliken charge between O10 and OY on ImPy along the reaction coordinate. (DOCX)

S12 Fig The ESD analysis on the geometries at the beginning of the biradical stage in Reaction_4 and in Reaction_3’. (DOCX)

S13 Fig Electron transfer within the dioxetanone intermediate in Reaction_4. (DOCX)

S14 Fig The S0 PESs of the cleavage of isolated anionic/protonated dioxetanone intermediate. (DOCX)

S1 Supplemental Structures Intact_GLuc_AF2.pdb. Docked_complex.pdb. Docked_complex_2nd.pdb. Docked_complex_3rd.pdb. Active_cluster_React.pdb.
(ZIP)
