# Supplementary material for: A Structural Model of Truncated Gaussia princeps Luciferase Elucidating the Crucial Catalytic Function of No.76 Arginine towards Coelenterazine Oxidation
Source: PLoS Comput Biol. 2025 Jan 21;21(1):e1012722. doi: 10.1371/journal.pcbi.1012722 (PMC11750096; doi:10.1371/journal.pcbi.1012722)
Supplement: S1 Fig — (DOCX) [file pcbi.1012722.s001.docx]

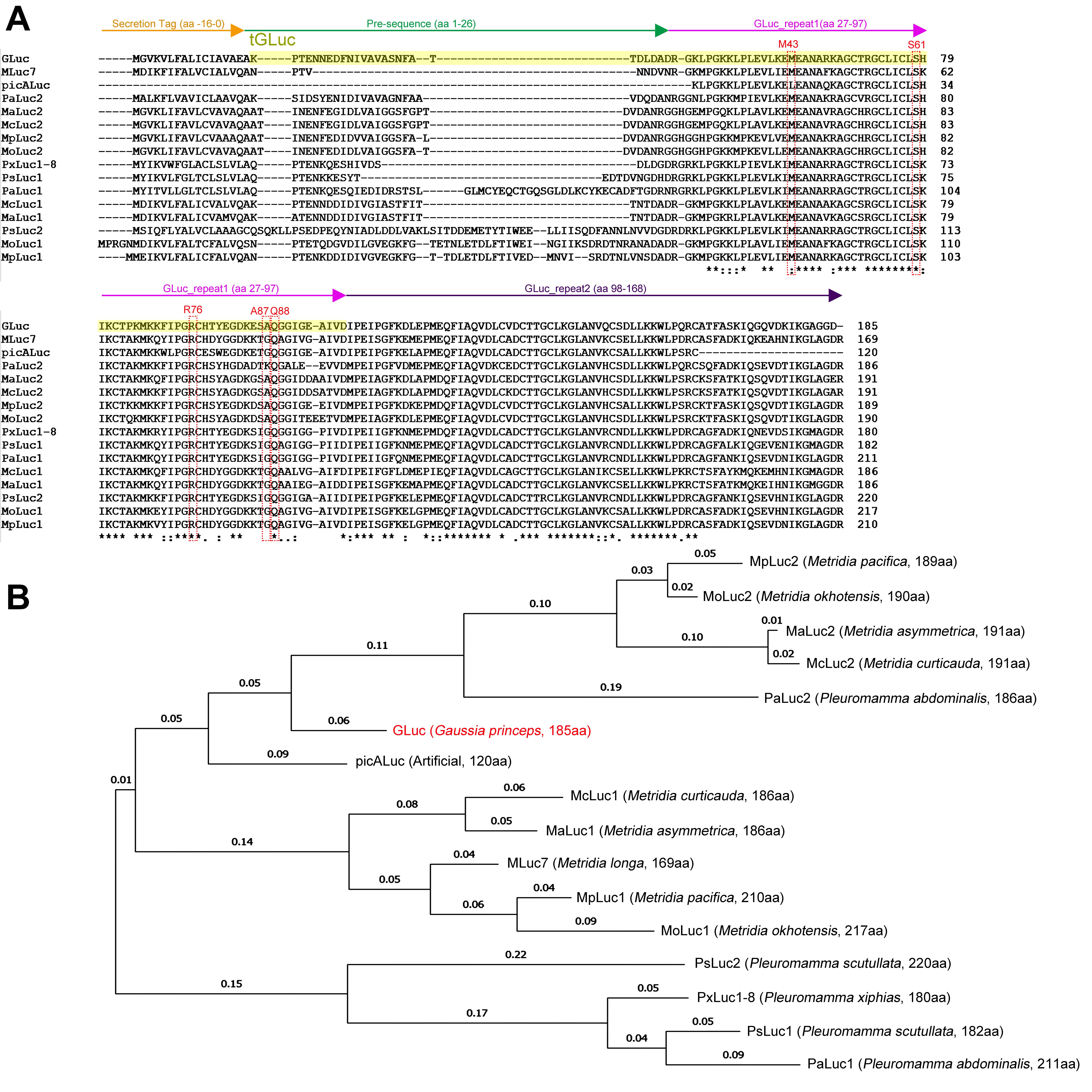


**S1 Fig.** (A) Sequence alignment of GLuc. A Basic Local Alignment Search Tool (BLAST) [1] search against the Swiss-Prot dataset UniprotKB identified 14 luciferases with significant sequence similarities and E-values below 10^-30^. picALuc, a synthetic luciferase [2], was not registered in the UniprotKB database, but was included here because it is the smallest CTZ luciferase and shares a similar sequence with GLuc. Amino acid conservation across the 15 sequences was indicated below the alignment: an asterisk denotes a fully conserved residue, a colon denotes a highly conserved residue, and a period denotes a poorly conserved residue, all of which were based on the Clustal W [3] classification. GLuc’s secretion tag, pre-sequence and two consecutive repeats were indicated above GLuc’s sequence. The sequence of tGLuc that comprises the pre-sequence and GLuc_repeat1 was highlighted by a yellow background. Amino acid residues M43, S61, R76, A87 and Q88 that were mentioned in this article were boxed. (B) Phylogenetic tree topology of GLuc and selected 15 similar luciferases constructed by maximum likelihood using MEGA11 [4]. GLuc is colored red. The origin and residue number are indicated. Branch length represents residue substitution per site.

**References**

1. Altschul, S. F., Gish, W., Miller, W., Myers, E. W. & Lipman, D. J. (1990). Basic local alignment search tool. J Mol Biol. **215**, 403-410.

2. Ohmuro-Matsuyama, Y., Matsui, H., Kanai, M. & Furuta, T. (2023). Glow-type conversion and characterization of a minimal luciferase via mutational analyses. FEBS J. **290**(23), 5554-5565.

3. Thompson, J. D., Higgins, D. G. & Gibson, T. J. (1994). CLUSTAL W: improving the sensitivity of progressive multiple sequence alignment through sequence weighting, position-specific gap penalties and weight matrix choice. Nucleic Acids Res. **22**, 4673-4680.

4. Tamura, K., Stecher, G. & Kumar, S. (2021). MEGA11: Molecular Evolutionary Genetics Analysis Version 11. Molecular Biology and Evolution. **38**, 3022-3027.
