# Supplementary material for: A Structural Model of Truncated Gaussia princeps Luciferase Elucidating the Crucial Catalytic Function of No.76 Arginine towards Coelenterazine Oxidation
Source: PLoS Comput Biol. 2025 Jan 21;21(1):e1012722. doi: 10.1371/journal.pcbi.1012722 (PMC11750096; doi:10.1371/journal.pcbi.1012722)
Supplement: S2 Fig — (DOCX) [file pcbi.1012722.s002.docx]

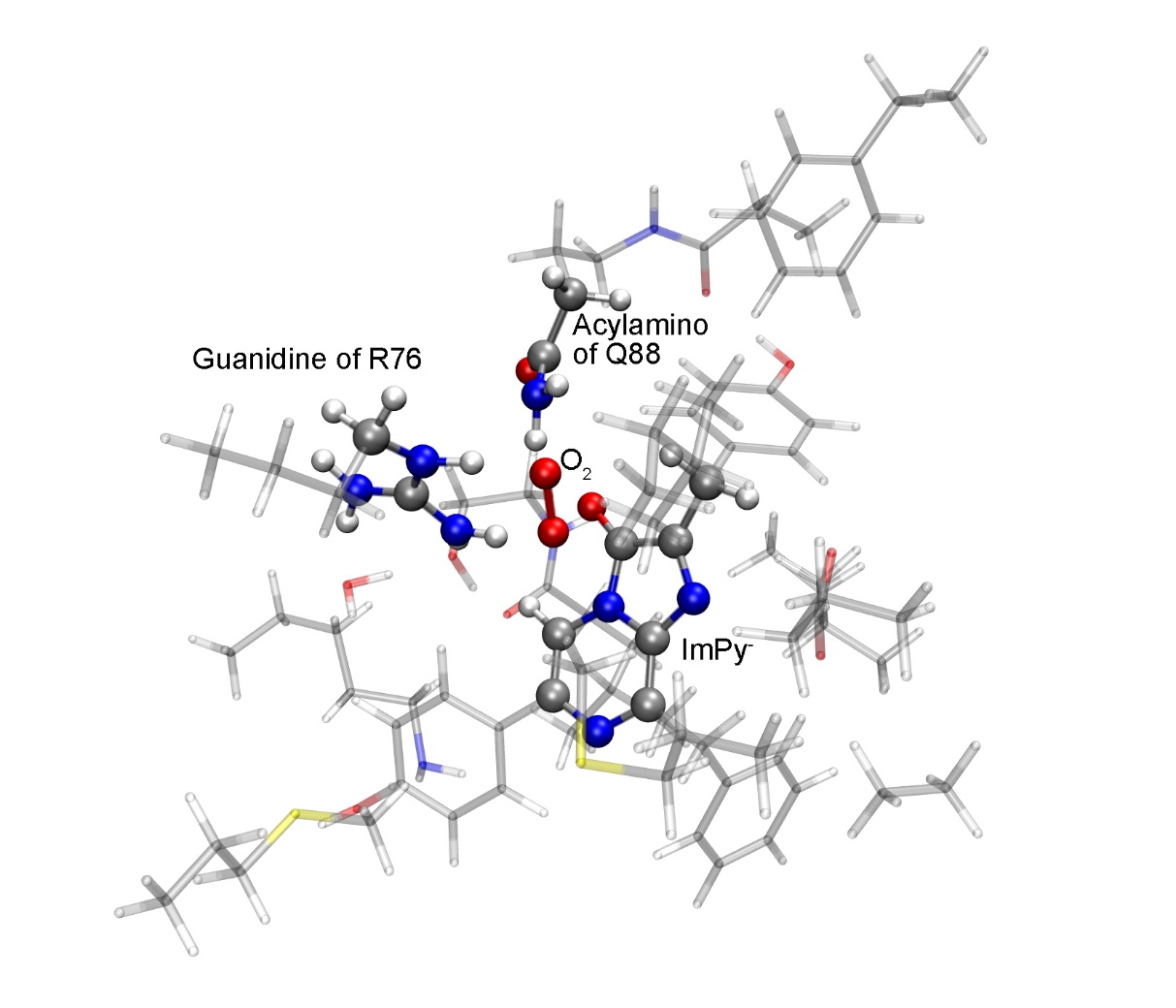


**S2 Fig.** The mixed basis set for the active_cluster. Atoms using the 6-31G(d,p) basis set were displayed in ball-and-stick model; Atoms using the 3-21G* basis set were displayed in transparent stick model.
