# Supplementary material for: A Structural Model of Truncated Gaussia princeps Luciferase Elucidating the Crucial Catalytic Function of No.76 Arginine towards Coelenterazine Oxidation
Source: PLoS Comput Biol. 2025 Jan 21;21(1):e1012722. doi: 10.1371/journal.pcbi.1012722 (PMC11750096; doi:10.1371/journal.pcbi.1012722)
Supplement: S3 Fig — (DOCX) [file pcbi.1012722.s003.docx]

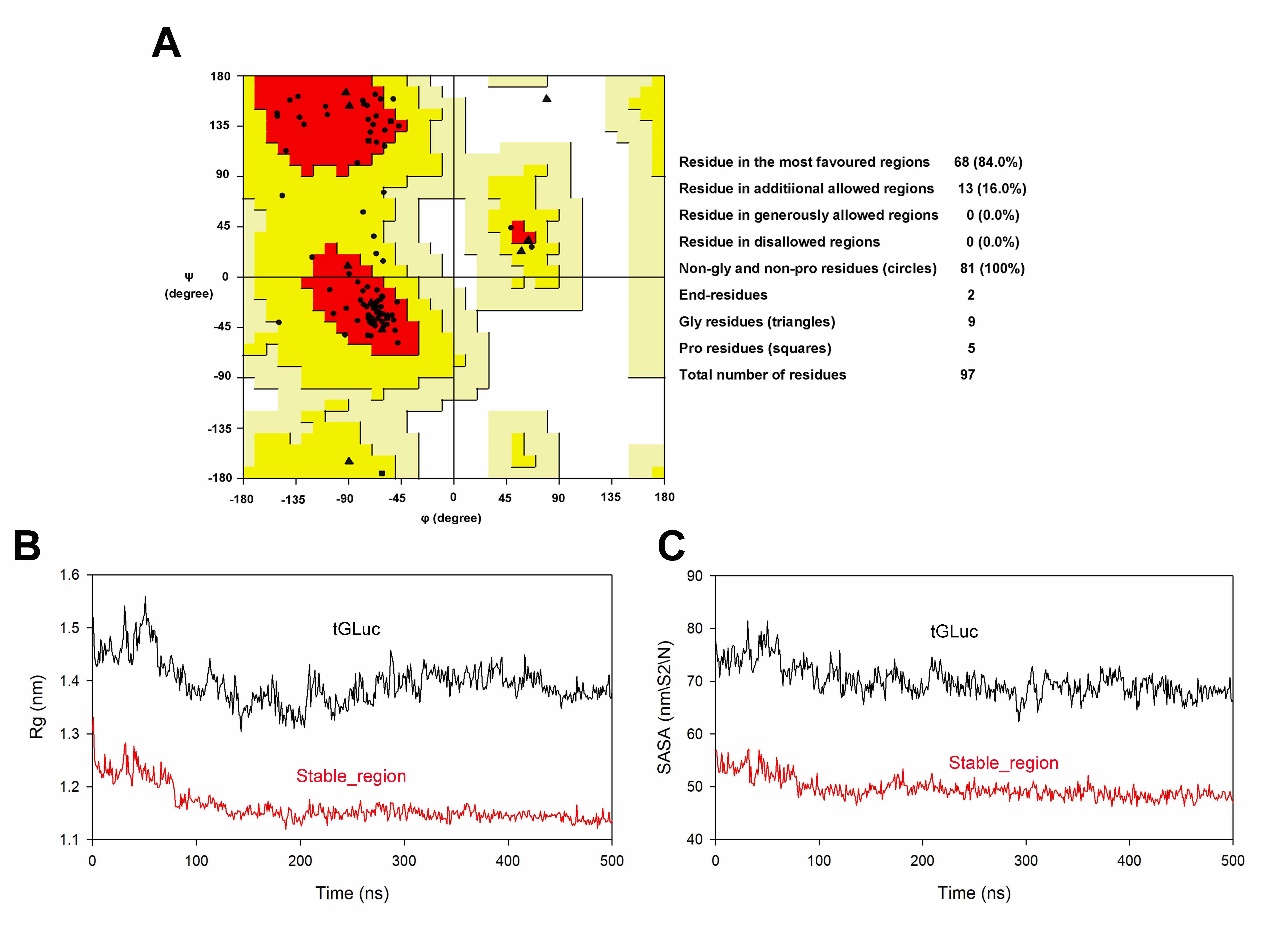


**S3 Fig.** (A) shows the Ramachandran plot of the representative structure identified through PCA analysis of the 500 ns simulation trajectory; (B) shows the time evolution of full-length tGLuc and its stable_region’s radius of gyration (Rg); and (C) shows the time evolution of full-length tGLuc and its stable_region’s solvent-accessible surface area (SASA) during the 500 ns MD simulation.
