# Supplementary material for: A Structural Model of Truncated Gaussia princeps Luciferase Elucidating the Crucial Catalytic Function of No.76 Arginine towards Coelenterazine Oxidation
Source: PLoS Comput Biol. 2025 Jan 21;21(1):e1012722. doi: 10.1371/journal.pcbi.1012722 (PMC11750096; doi:10.1371/journal.pcbi.1012722)
Supplement: S4 Fig — (DOCX) [file pcbi.1012722.s004.docx]

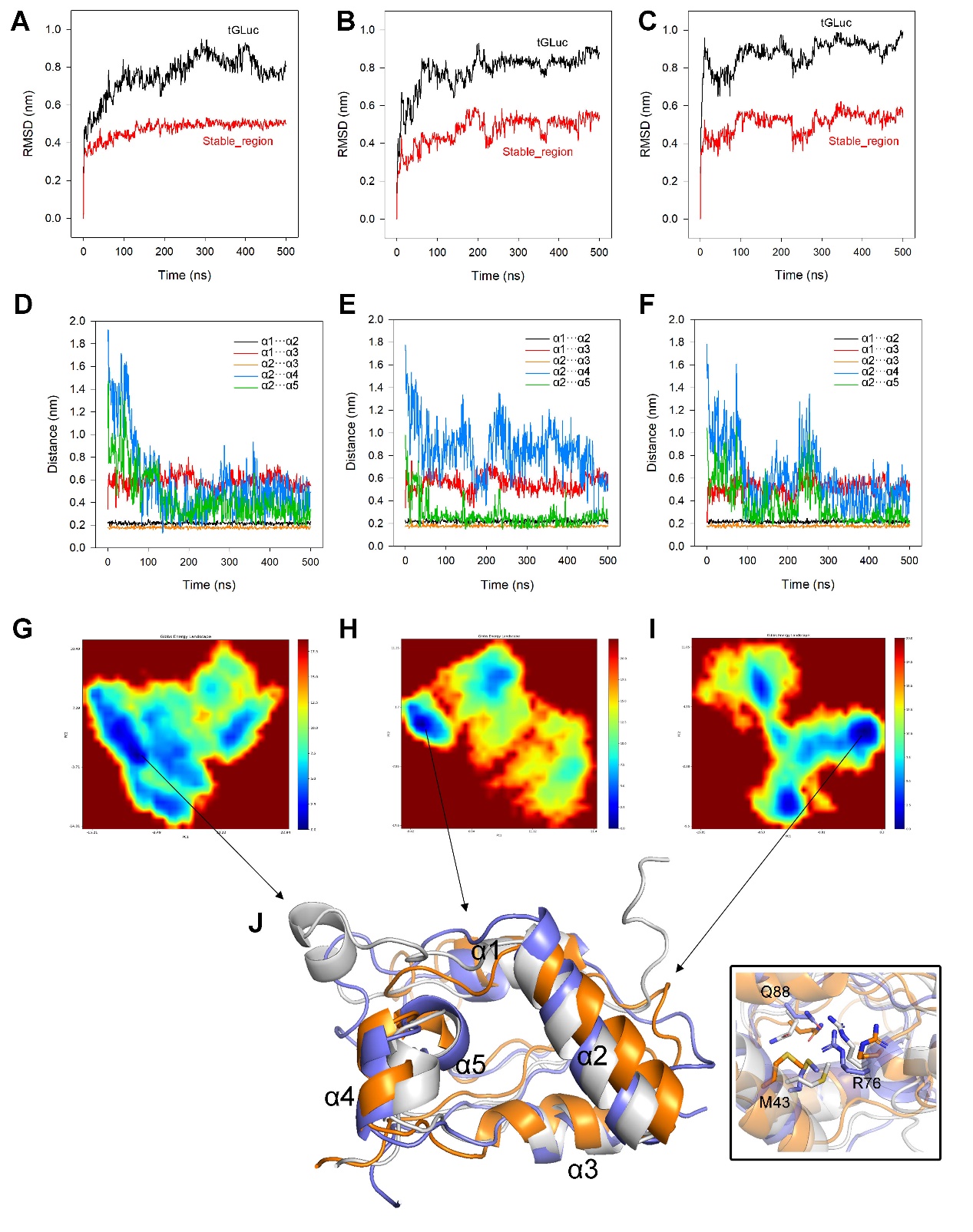


**S4 Fig.** Three independent 500 ns MD simulations starting from the AF2 tGLuc structure with randomized initial velocities (each column corresponds to the same simulation, where the first column (A)(D)(G) refers to the trajectory analyzed in the main text and S3 Fig). Panels (A)-(C) show the time evolution of RMSD between full-length tGLuc and stable_region over time for the three simulations; panels (D)-(F) illustrate the changes in α-helix spacing of tGLuc over time; and panels (G)-(I) depict free energy landscapes obtained from PCA analysis of the three MD simulation trajectories. Panel (J) shows the superimposed structures with the lowest energy in each landscape (first column: white; second column: blue; third column: yellow). The RMSD between the stable_regions are consistently below 0.25 nm, and the inserted figure on the right illustrates the positions of M43, which affects the full-length GLuc activity [1-4], and R76 [5, 6] and Q88, which influence ImPy oxidation in subsequent DFT calculations.

**References**

1. Maguire, C. A., Deliolanis, N. C., Pike, L., Niers, J. M., Tjon-Kon-Fat, L. A., Sena-Esteves, M. & Tannous, B. A. (2009). *Gaussia* luciferase variant for high-throughput functional screening applications. Anal Chem. **81**, 7102-7106.

2. Welsh, J. P., Patel, K. G., Manthiram, K. & Swartz, J. R. (2009). Multiply mutated *Gaussia* luciferases provide prolonged and intense bioluminescence. Biochem Biophys Res Commun. **389**, 563-568.

3. Degeling, M. H., Bovenberg, M. S., Lewandrowski, G. K., de Gooijer, M. C., Vleggeert-Lankamp, C. L., Tannous, M., Maguire, C. A. & Tannous, B. A. (2013). Directed molecular evolution reveals *Gaussia* luciferase variants with enhanced light output stability. Anal Chem. **85**, 3006-3012.

4. Sun, M., Fu, Z., Wang, T., Cui, X., Dong, J., Du, F., Huang, X., Lu, X., Chen, G., Sun, J., Wang, C. & Tang, Z. (2018). A high-throughput in vivo selection method for luciferase variants. Sensors and Actuators B: Chemical. **273**, 191-197.

5. Kim, S. B., Suzuki, H., Sato, M. & Tao, H. (2011). Superluminescent variants of marine luciferases for bioassays. Anal Chem. **83**, 8732-8740.

6. Dijkema, F. M., Escarpizo-Lorenzana, M. I., Nordentoft, M. K., Rabe, H. C., Sahin, C., Landreh, M., Branca, R. M., Sørensen, E. S., Christensen, B., Prestel, A., Teilum, K. & Winther, J. R. (2024). A suicidal and extensively disordered luciferase with a bright luminescence. Protein Science. **33**, e5115.
