# Supplementary material for: A Structural Model of Truncated Gaussia princeps Luciferase Elucidating the Crucial Catalytic Function of No.76 Arginine towards Coelenterazine Oxidation
Source: PLoS Comput Biol. 2025 Jan 21;21(1):e1012722. doi: 10.1371/journal.pcbi.1012722 (PMC11750096; doi:10.1371/journal.pcbi.1012722)
Supplement: S5 Fig — (DOCX) [file pcbi.1012722.s005.docx]

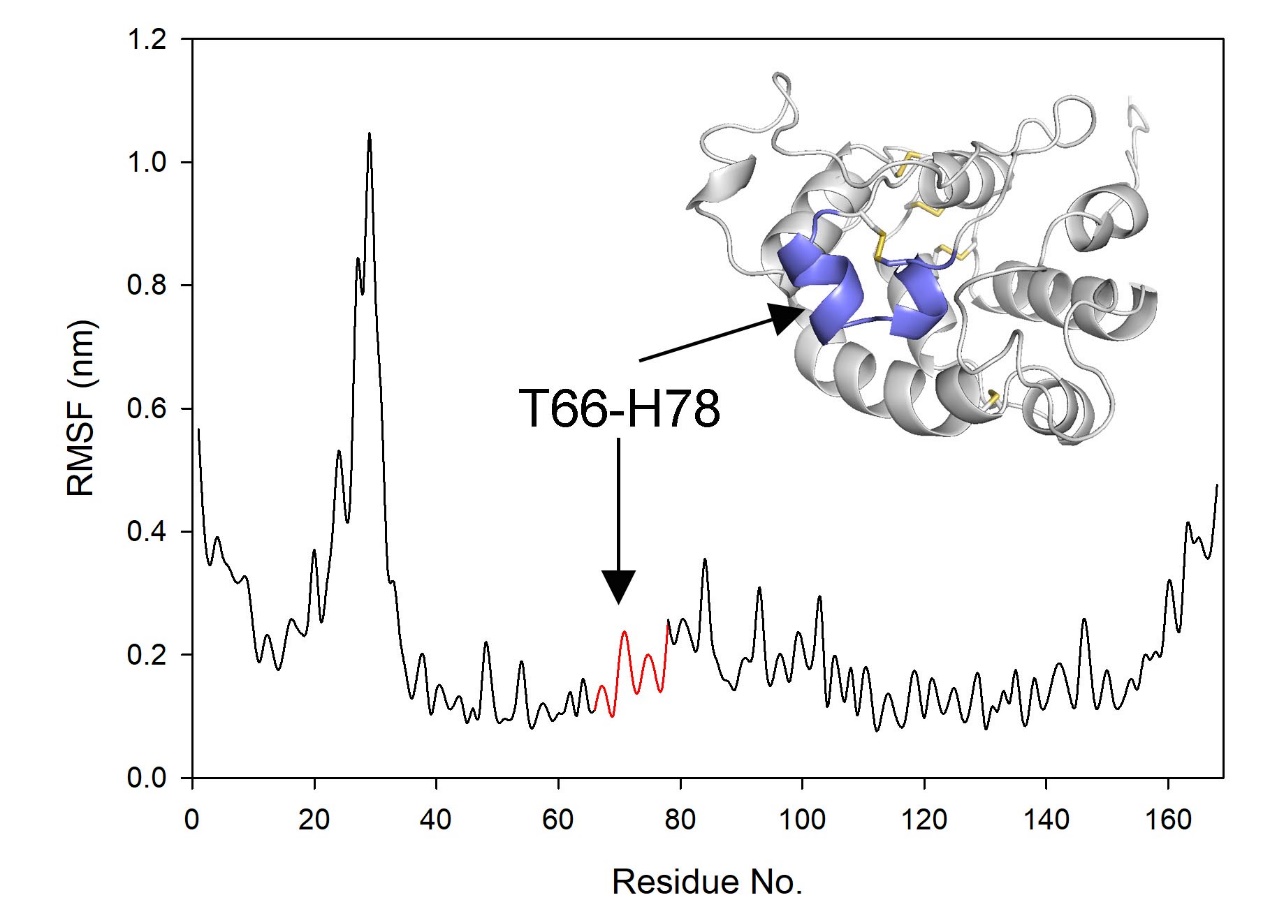


**S5 Fig.** RMSF of the 200 ns MD simulation trajectory of full-length GLuc (AF2 structure, with aa 1-168). The simulation conditions are consistent with those described in section 2.2.1. The RMSF of the T66-H78 region (in red) and its position within full-length GLuc are marked (in blue).
