# Supplementary material for: A Structural Model of Truncated Gaussia princeps Luciferase Elucidating the Crucial Catalytic Function of No.76 Arginine towards Coelenterazine Oxidation
Source: PLoS Comput Biol. 2025 Jan 21;21(1):e1012722. doi: 10.1371/journal.pcbi.1012722 (PMC11750096; doi:10.1371/journal.pcbi.1012722)
Supplement: S6 Fig — (DOCX) [file pcbi.1012722.s006.docx]

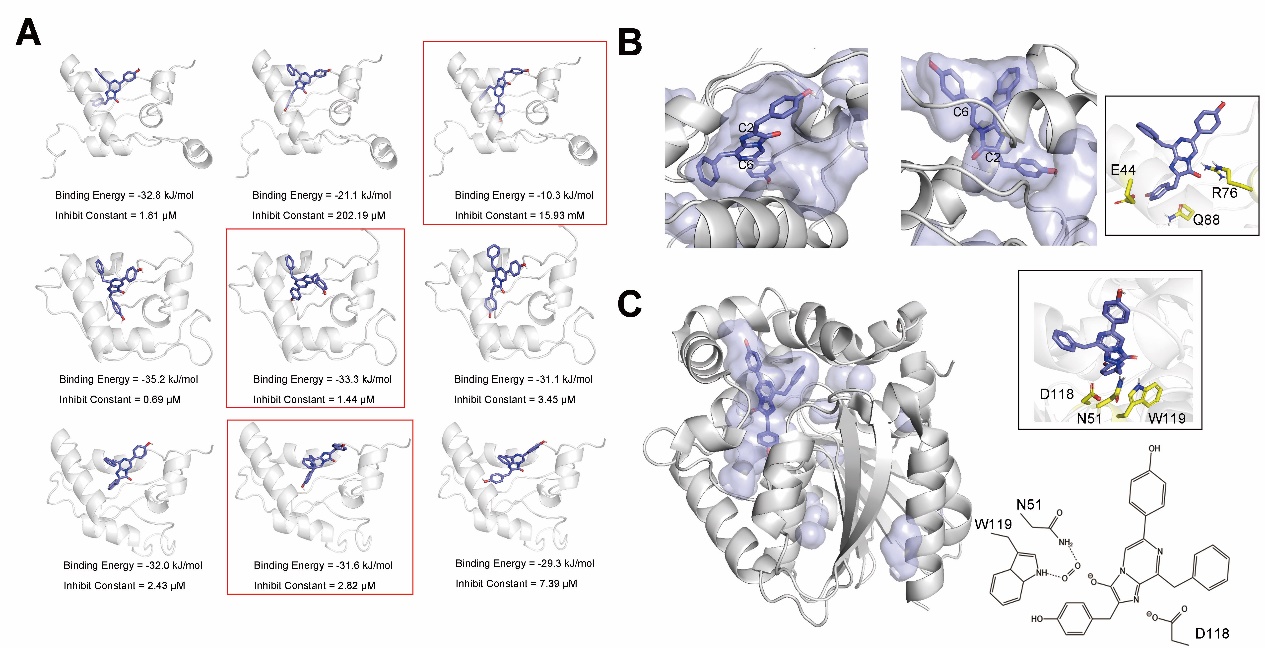


**S6 Fig.** Molecular docking of tGLuc with CTZ using Autodock4[1]. Three docking runs were conducted using three representative structures of tGLuc (S4J Fig) as receptors and CTZ as the ligand. Each run produced one hundred tGLuc-CTZ complex conformations, from which the three with the lowest binding energy were selected, resulting in a total of nine docked complexes. (A) displays these nine selected docking complexes, with each row corresponding to one docking run. The coordinate files of the leftmost structures (with the lowest binding energy in each run) in the three rows are provided in Supplemental_Structures/ Docked_complex.pdb, Docked_complex_2nd.pdb, and Docked_complex_3rd.pdb. Further classification of the nine conformations revealed two poses of CTZ within the tGLuc cavity. Pose_1 conformations are highlighted in red boxes, while the remaining conformations belong to pose_2. (B) illustrates the differences between pose_1 and pose_2 using the rightmost and leftmost structures in row 1 of (A). The left panel in (B) shows pose_1, where the C6G of CTZ faces the catalytic cavity interior, and C2G faces the cavity entrance. In contrast, the right panel shows pose_2, where C2G faces the catalytic cavity interior, and C6G faces the cavity entrance. We propose that CTZ adopts pose_2 within tGLuc for the following reasons: (1) The catalytic mechanism of CTZ as a substrate has been elucidated for *Renilla* luciferase[2], Obelin[3], both demonstrating that CTZ, particularly the C2 atom on its initial oxygenation site ImPy, resides in a highly hydrophobic environment within the catalytic cavity, facilitating its attack by oxygen molecules to form 2-proxy-CTZ. In pose_1, the C2 atom faces the cavity entrance and is easily accessed by water, whereas in pose_2, the C2 atom faces the catalytic cavity interior, ensuring a hydrophobic environment. (2) In 2024, Schenkmayerova et al. resolved the co-crystal structure of *Renilla* luciferase with the inactive CTZ analog aza-CTZ (left panel in (C)), and demonstrated that N51, D118, and W119 located near the aza-CTZ are activity-associated residues (right panel in (C)). The sketch in the bottom right panel illustrates Schenkmayerova et al’s speculation that W119 and N51 bind an oxygen molecule, facilitating its nucleophilic attack on the C2 of ImPy, while D118 plays a role in proton transfer[2]. The rightmost inset of (B) shows the proximity of R76, Q88, and E44 of tGLuc to CTZ in pose_2, closely resembling the RLuc catalytic center. Thus, we infer that CTZ adopts pose_2 within tGLuc, ultimately selecting the upper left conformation in (A) for subsequent calculations.

**References**

1. Morris, G. M., Huey, R., Lindstrom, W., Sanner, M. F., Belew, R. K., Goodsell, D. S. & Olson, A. J. (2009). AutoDock4 and AutoDockTools4: Automated docking with selective receptor flexibility. Journal of Computational Chemistry. **30**, 2785-2791.

2. Schenkmayerova, A., Toul, M., Pluskal, D., Baatallah, R., Gagnot, G., Pinto, G. P., Santana, V. T., Stuchla, M., Neugebauer, P., Chaiyen, P., Damborsky, J., Bednar, D., Janin, Y. L., Prokop, Z. & Marek, M. (2023). Catalytic mechanism for *Renilla*-type luciferases. Nature Catalysis. **6**, 23-38.

3. Vysotski, E. S., Liu, Z.-J., Markova, S. V., Blinks, J. R., Deng, L., Frank, L. A., Herko, M., Malikova, N. P., Rose, J. P., Wang, B. C. & Lee, J. (2003). Violet bioluminescence and fast kinetics from W92F obelin:  Structure-based proposals for the bioluminescence triggering and the identification of the emitting species. Biochemistry. **42**, 6013-6024.
