# Supplementary material for: A Structural Model of Truncated Gaussia princeps Luciferase Elucidating the Crucial Catalytic Function of No.76 Arginine towards Coelenterazine Oxidation
Source: PLoS Comput Biol. 2025 Jan 21;21(1):e1012722. doi: 10.1371/journal.pcbi.1012722 (PMC11750096; doi:10.1371/journal.pcbi.1012722)
Supplement: S7 Fig — (DOCX) [file pcbi.1012722.s007.docx]

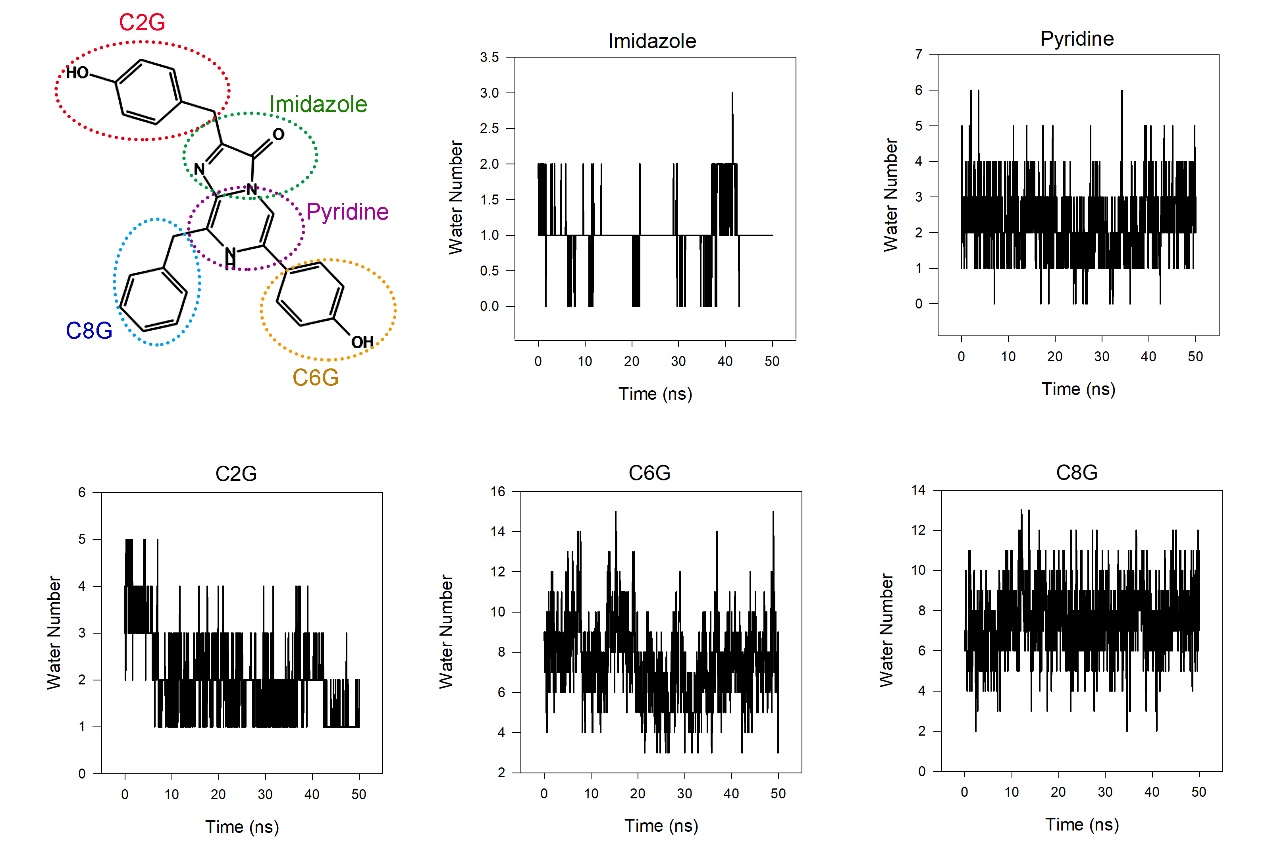


**S7 Fig.** The number of water molecules within a radius of 3.5 Å around the Imidazole, Pyridine, C2G, C6G and C8G of CTZ during a 50ns GLuc-CTZ MD simulation that introduced ten O_2_ molecules in the system.
