# Supplementary material for: A Structural Model of Truncated Gaussia princeps Luciferase Elucidating the Crucial Catalytic Function of No.76 Arginine towards Coelenterazine Oxidation
Source: PLoS Comput Biol. 2025 Jan 21;21(1):e1012722. doi: 10.1371/journal.pcbi.1012722 (PMC11750096; doi:10.1371/journal.pcbi.1012722)
Supplement: S8 Fig — (DOCX) [file pcbi.1012722.s008.docx]

**
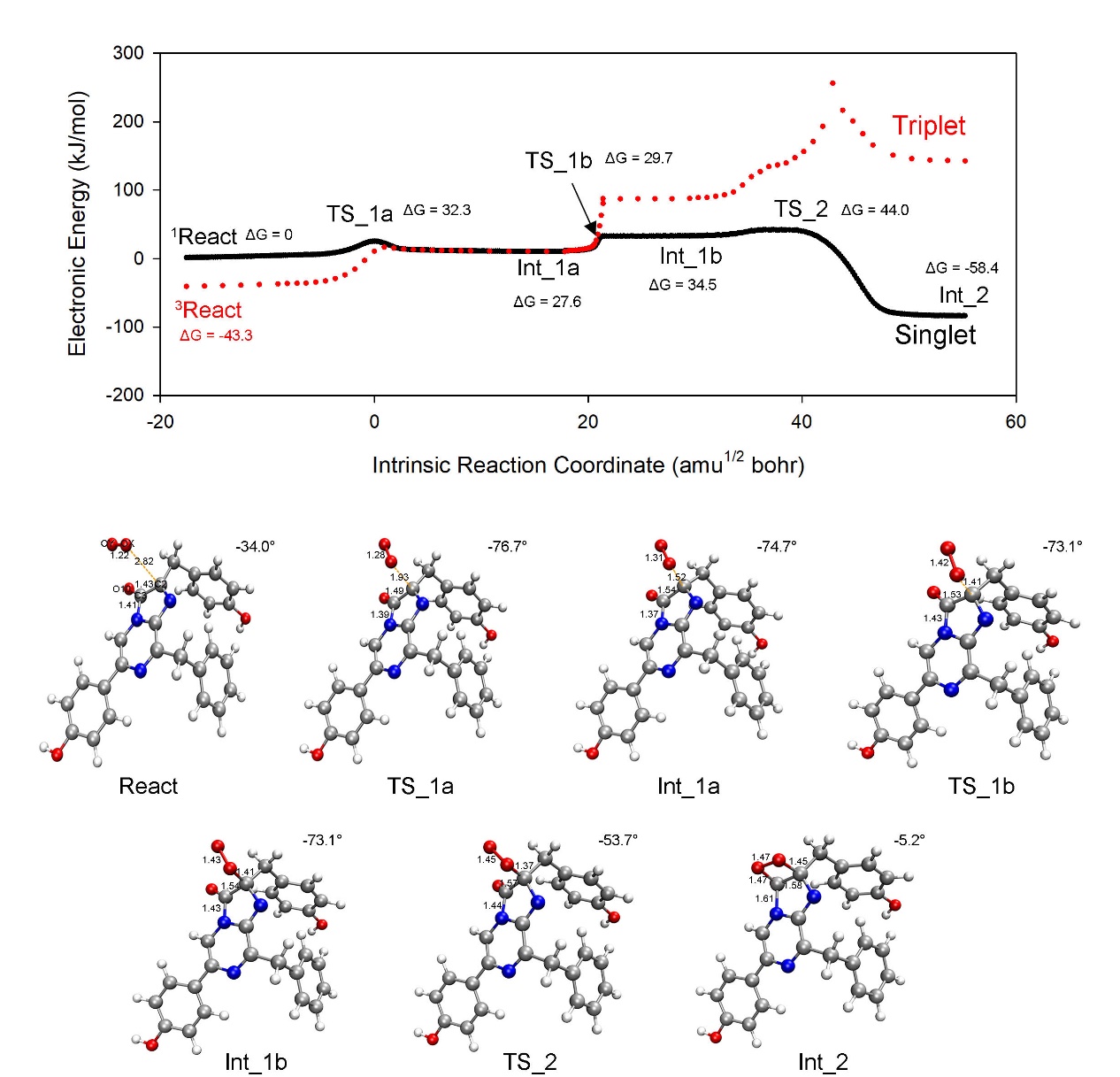
**

**S8 Fig.** Singlet/triplet PES of the isolated CTZ oxidation begin with React (with spin multiplicity marked on the upper left) to the formation of Int_2 dioxetanone^-^. The singlet and triplet PES are depicted in black and red, respectively, with the structures at each stationary point displayed below. Key interatomic distances are labeled, with the dihedral angle of C3-C2-OX-OY noted in the upper right. The singlet PES indicates that, without considering catalytic residues, oxygen addition to ImPy proceeds through two elementary steps involving transition states TS_1a and TS_1b, corresponding to Reaction_1 in the main text under catalytic conditions. The subsequent transition state, TS_2, corresponds to the formation of dioxetanone, as described in Reaction_2 in the main text. Under natural conditions, the reaction initiates from triplet state ^3^React, with ISC to the singlet state at Int_1a (ΔG barrier = 70.9 kJ/mol), after which the reaction energy continue to increase until TS_2. Thus, the overall ΔG barrier, up to dioxetanone formation, should be the energy difference between TS_2 and ^3^React, which is 87.3 kJ/mol.
