# Supplementary figures and images for: A Structural Model of Truncated Gaussia princeps Luciferase Elucidating the Crucial Catalytic Function of No.76 Arginine towards Coelenterazine Oxidation
Source: PLoS Comput Biol. 2025 Jan 21;21(1):e1012722. doi: 10.1371/journal.pcbi.1012722 (PMC11750096; doi:10.1371/journal.pcbi.1012722)

**
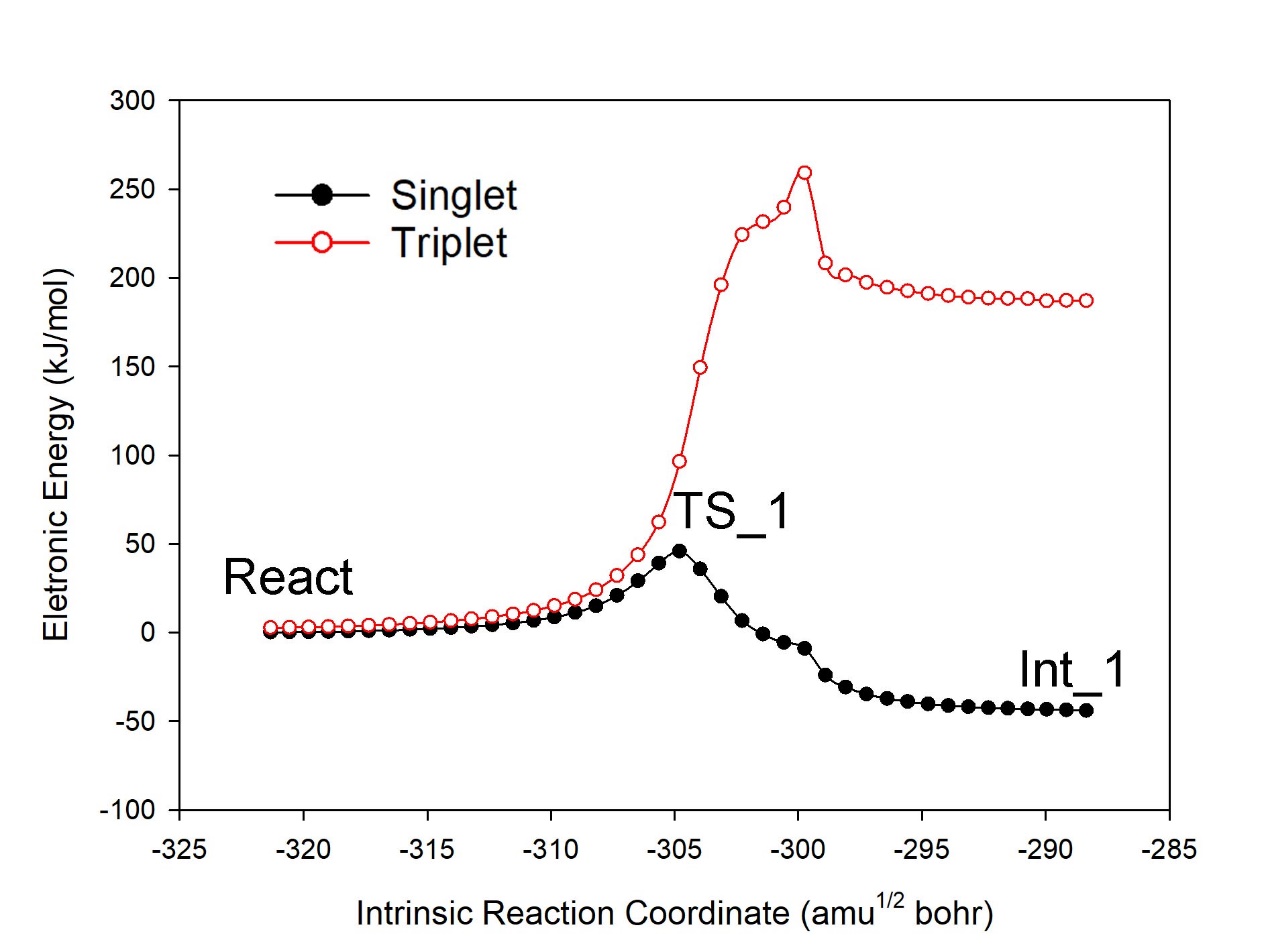
**

**S9 Fig.** Singlet/triplet PES of the Reaction_1

Supplement: S9 Fig — (DOCX) [file pcbi.1012722.s009.docx]
