# Supplementary material for: A Structural Model of Truncated Gaussia princeps Luciferase Elucidating the Crucial Catalytic Function of No.76 Arginine towards Coelenterazine Oxidation
Source: PLoS Comput Biol. 2025 Jan 21;21(1):e1012722. doi: 10.1371/journal.pcbi.1012722 (PMC11750096; doi:10.1371/journal.pcbi.1012722)
Supplement: S10 Fig — (DOCX) [file pcbi.1012722.s010.docx]

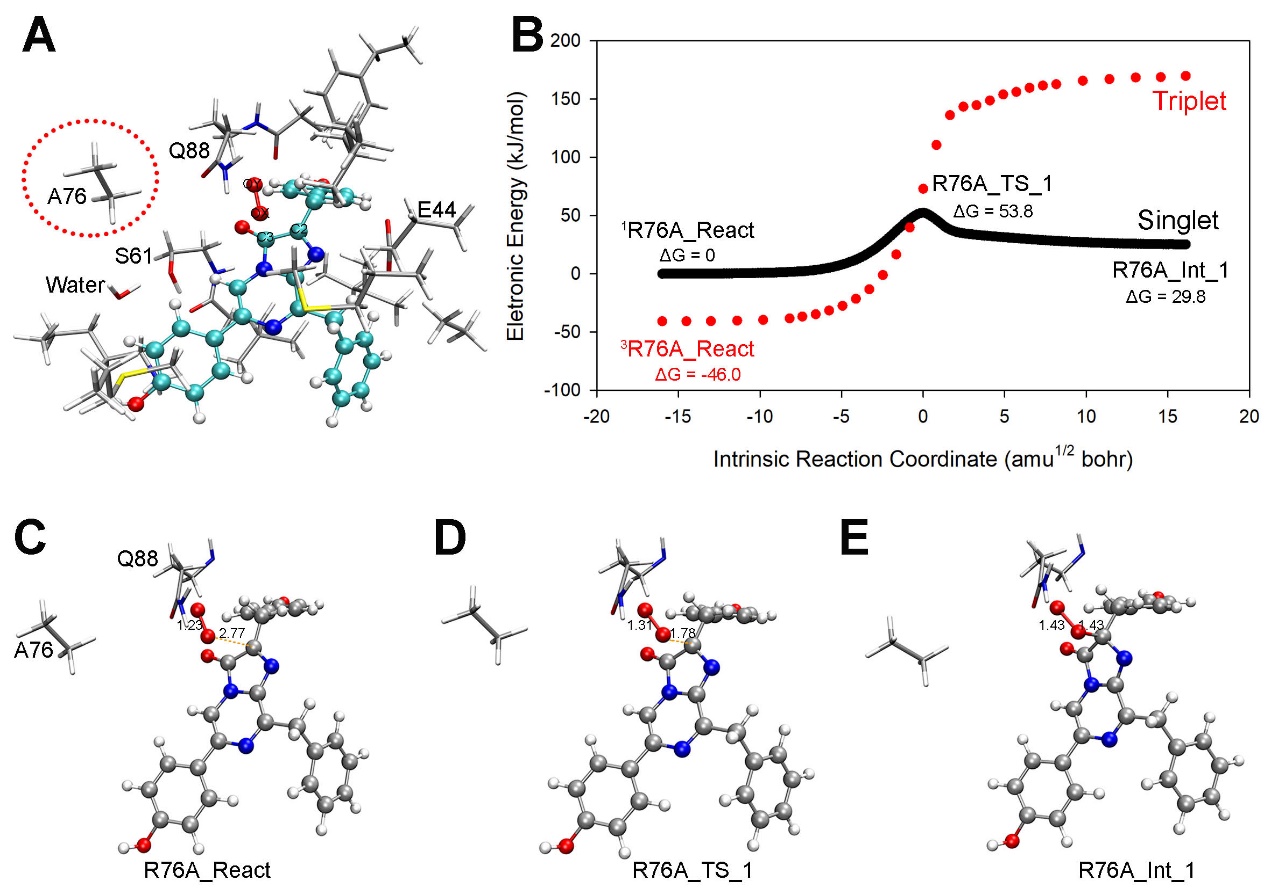


**S10 Fig.** Singlet/triplet PES of Reaction_1 within alanine substitution on the R76 (R76A). (A) shows the active_cluster incorporating the R76A (constructed by using the structure of Fig 6F as a template); (B) singlet/triplet PES of Reaction_1 with R76A, with the initial reactant being R76A_React (spin multiplicity was marked on the upper left), the transition state R76A_TS_1, and the intermediate R76A_Int_1. The relative positions of proxy-CTZ and nearby residues Q88 and A76 are shown in (C)-(E). Without the catalytic influence of R76, the ΔG gap between the ^3^React and ^1^React is 46 kJ/mol. During Reaction_1, beginning with ^3^React to the R76A_TS_1, the oxygen addition ΔG barrier reaches up to 98.8 kJ/mol, significantly higher than the ΔG barrier induced by R76 (65.4 kJ/mol, see Fig 7).
