# Supplementary material for: A Structural Model of Truncated Gaussia princeps Luciferase Elucidating the Crucial Catalytic Function of No.76 Arginine towards Coelenterazine Oxidation
Source: PLoS Comput Biol. 2025 Jan 21;21(1):e1012722. doi: 10.1371/journal.pcbi.1012722 (PMC11750096; doi:10.1371/journal.pcbi.1012722)
Supplement: S11 Fig — (DOCX) [file pcbi.1012722.s011.docx]

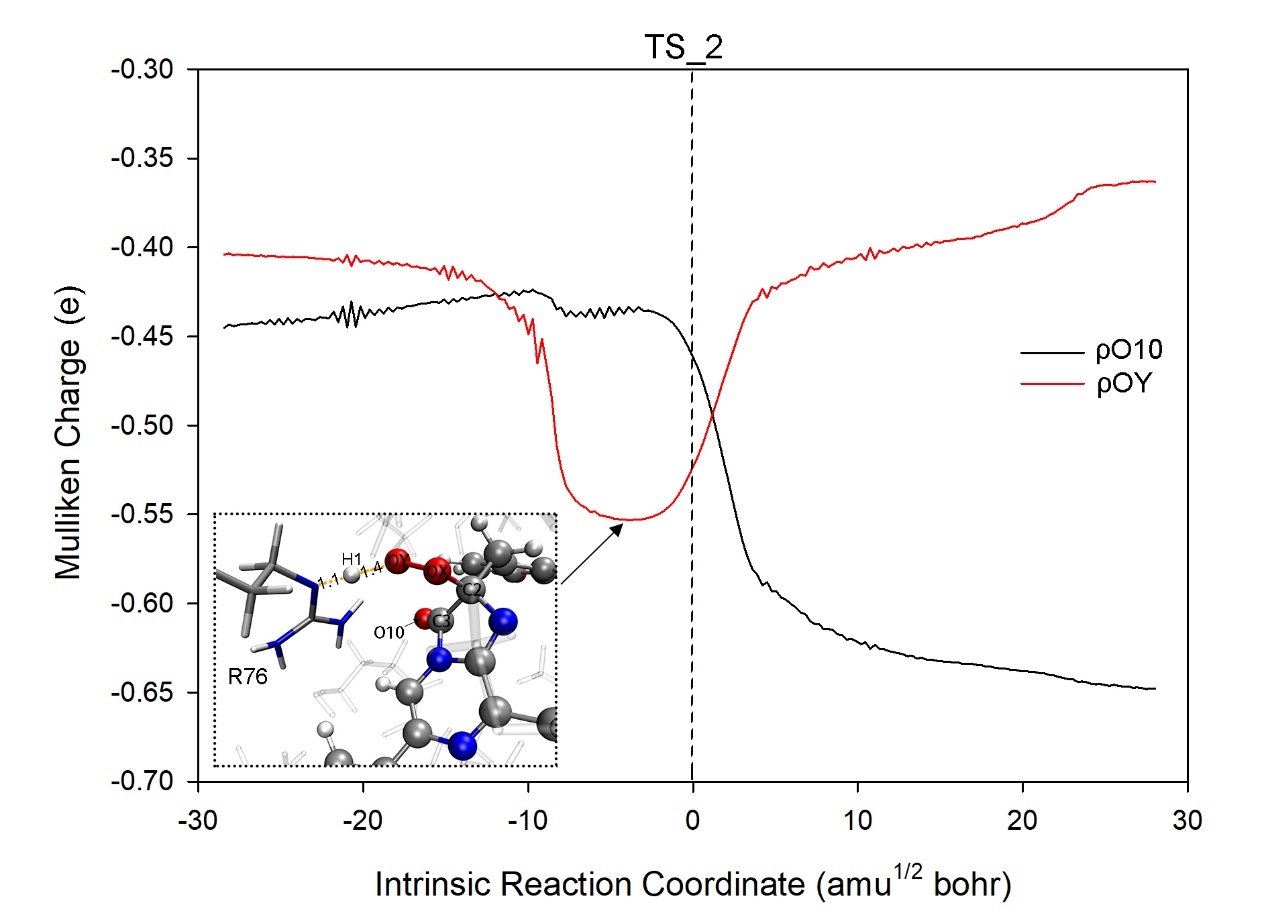


**S11 Fig.** The changes of Mulliken charge between O10 and OY on ImPy along the reaction coordinate. The geometries of ImPy and R76 at the charge minimum of OY (proton H1 was detached from OY and approached R76) were shown in the inset figure.
