# Supplementary material for: A Structural Model of Truncated Gaussia princeps Luciferase Elucidating the Crucial Catalytic Function of No.76 Arginine towards Coelenterazine Oxidation
Source: PLoS Comput Biol. 2025 Jan 21;21(1):e1012722. doi: 10.1371/journal.pcbi.1012722 (PMC11750096; doi:10.1371/journal.pcbi.1012722)
Supplement: S12 Fig — (DOCX) [file pcbi.1012722.s012.docx]

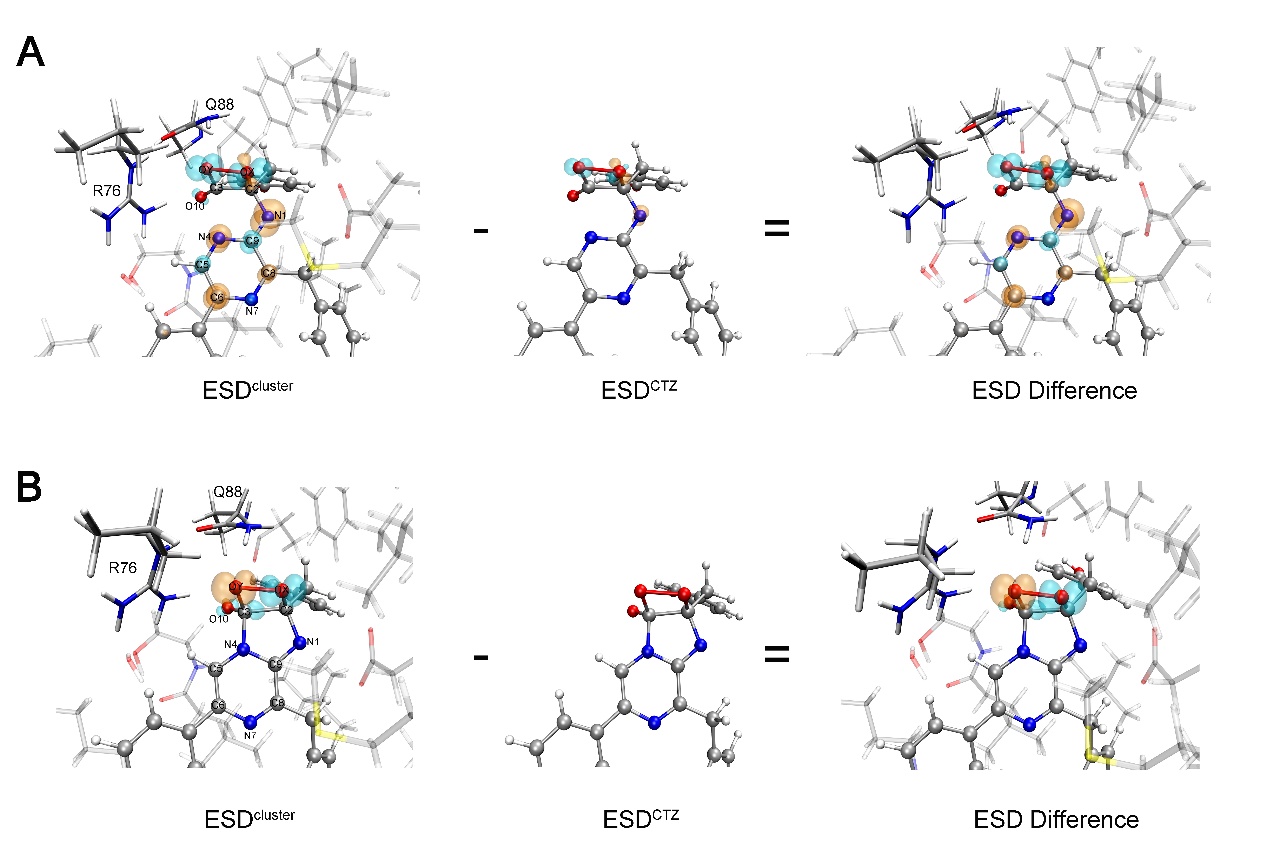


**S12 Fig.** The ESD analysis on the geometries at the beginning of the biradical stage in Reaction_4 (A) and in Reaction_3’ (B). The ESD^cluster^ represents the ESD analysis performed with active_cluster, The ESD^CTZ^ represents the ESD analysis performed only with the oxygenated CTZ, and ESD Difference was calculated using ESD^cluster^ - ESD^CTZ^. The regions with high α and β electron densities are indicated by transparent orange and cyan, respectively.
