# Supplementary material for: A Structural Model of Truncated Gaussia princeps Luciferase Elucidating the Crucial Catalytic Function of No.76 Arginine towards Coelenterazine Oxidation
Source: PLoS Comput Biol. 2025 Jan 21;21(1):e1012722. doi: 10.1371/journal.pcbi.1012722 (PMC11750096; doi:10.1371/journal.pcbi.1012722)
Supplement: S13 Fig — (DOCX) [file pcbi.1012722.s013.docx]

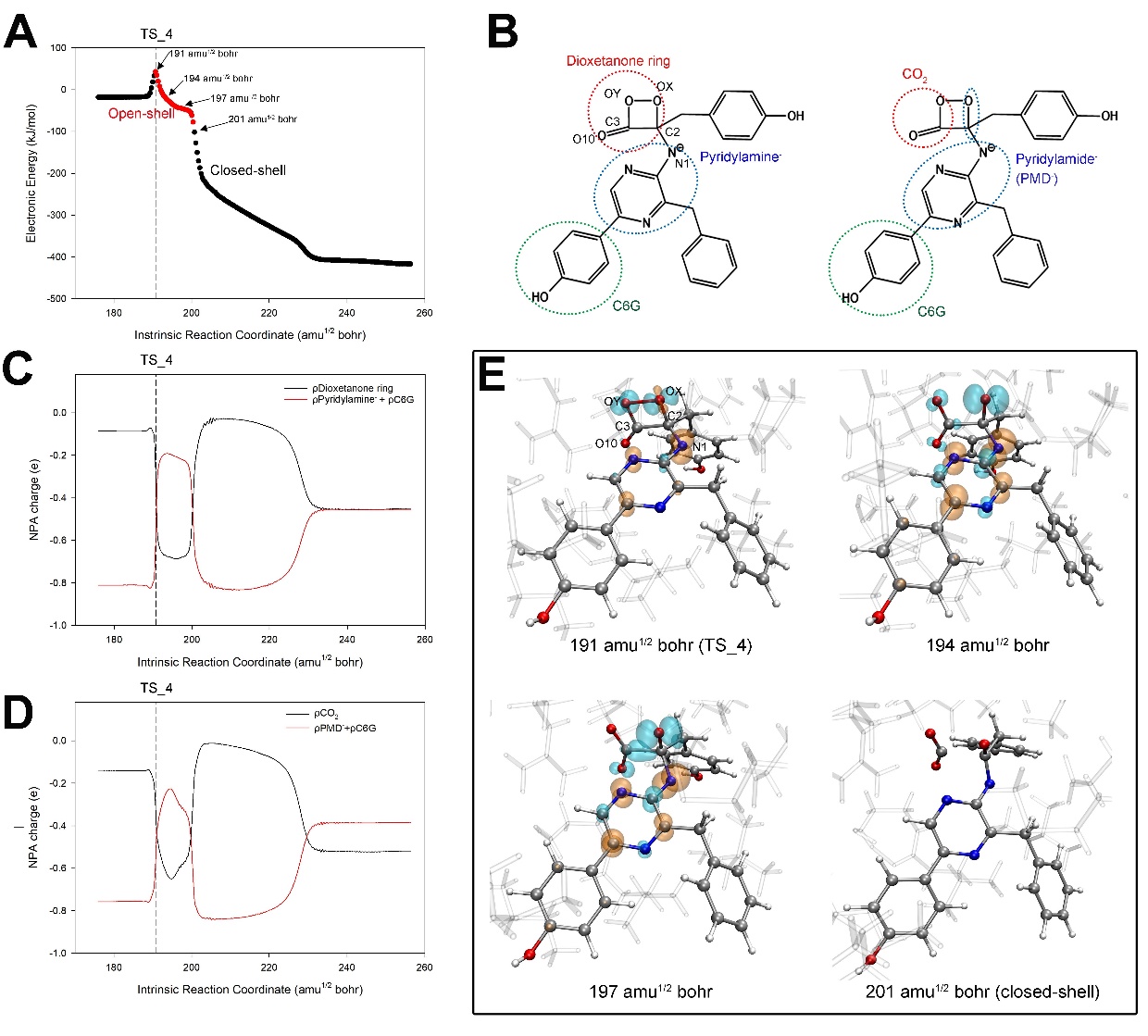


**S13 Fig.** Electron transfer within the dioxetanone intermediate in Reaction_4. Natural Population Analysis (NPA) charges were used to accurately describe charge changes. (A) shows the PES of Reaction_4, with open-shell denoted in red and closed-shell in black; (B) Schematic of the fragment range for NPA charge analysis within the dioxetanone intermediate; (C) NPA charge transfer between the dioxetanone ring and pyridylamine^-^ + C6G; (D) NPA charge transfer between CO_2_ and PMD^-^ + C6G (same as Fig 12E, but using the NPA charge instead of the Mulliken charge. Despite differences in the transferred charge amount, the transfer pattern is consistent between Mulliken and NPA charges); (E) ESD analysis of four nodes (marked in A) during the dioxetanone intermediate cleavage. The regions with high α and β electron densities are indicated by transparent orange and cyan, respectively.

From (C), near TS_4 (191 amu^1/2^ bohr), electrons from pyridylamine^-^ are transferred to the dioxetanone ring, resulting in α and β electrons being primarily distributed between pyridylamine^-^ and the dioxetanone ring, respectively (see E, top left, 191 amu^1/2^ bohr), corresponding to the ET step in Fig 11. (D) shows from 191 amu^1/2^ bohr to 194 amu^1/2^ bohr, the β electrons accumulating in the dioxetanone ring are increasingly concentrated in CO_2_, reaching a maximum at 194 amu^1/2^ bohr where OX-OY completely breaks (see E, top right, 194 amu^1/2^ bohr). From 194 amu^1/2^ bohr to 199 amu^1/2^ bohr, β electrons in CO_2_ are transferred via the C3-C2 bond to PMD^-^ (see E, bottom left, 197 amu^1/2^ bohr). (C) indicates that after 199 amu^1/2^ bohr, β electrons flow into pyridylamine^-^, ultimately resulting in the disappearance of free radical electrons at 201 amu^1/2^ bohr and the C2-C3 breakage (see E, bottom right, 201 amu^1/2^ bohr), corresponding to the BET step in Fig 11. Therefore, the described electron transfer process strictly adheres to the CIEEL theory. Additionally, (D) shows that at 233 amu^1/2^ bohr, charges between CO_2_ and PMD^-^ intersect again, and (A) indicates no free radical electrons at this point, implying mere charge transfer and not a CIEEL reaction process.
