# Supplementary material for: A Structural Model of Truncated Gaussia princeps Luciferase Elucidating the Crucial Catalytic Function of No.76 Arginine towards Coelenterazine Oxidation
Source: PLoS Comput Biol. 2025 Jan 21;21(1):e1012722. doi: 10.1371/journal.pcbi.1012722 (PMC11750096; doi:10.1371/journal.pcbi.1012722)
Supplement: S14 Fig — (DOCX) [file pcbi.1012722.s014.docx]

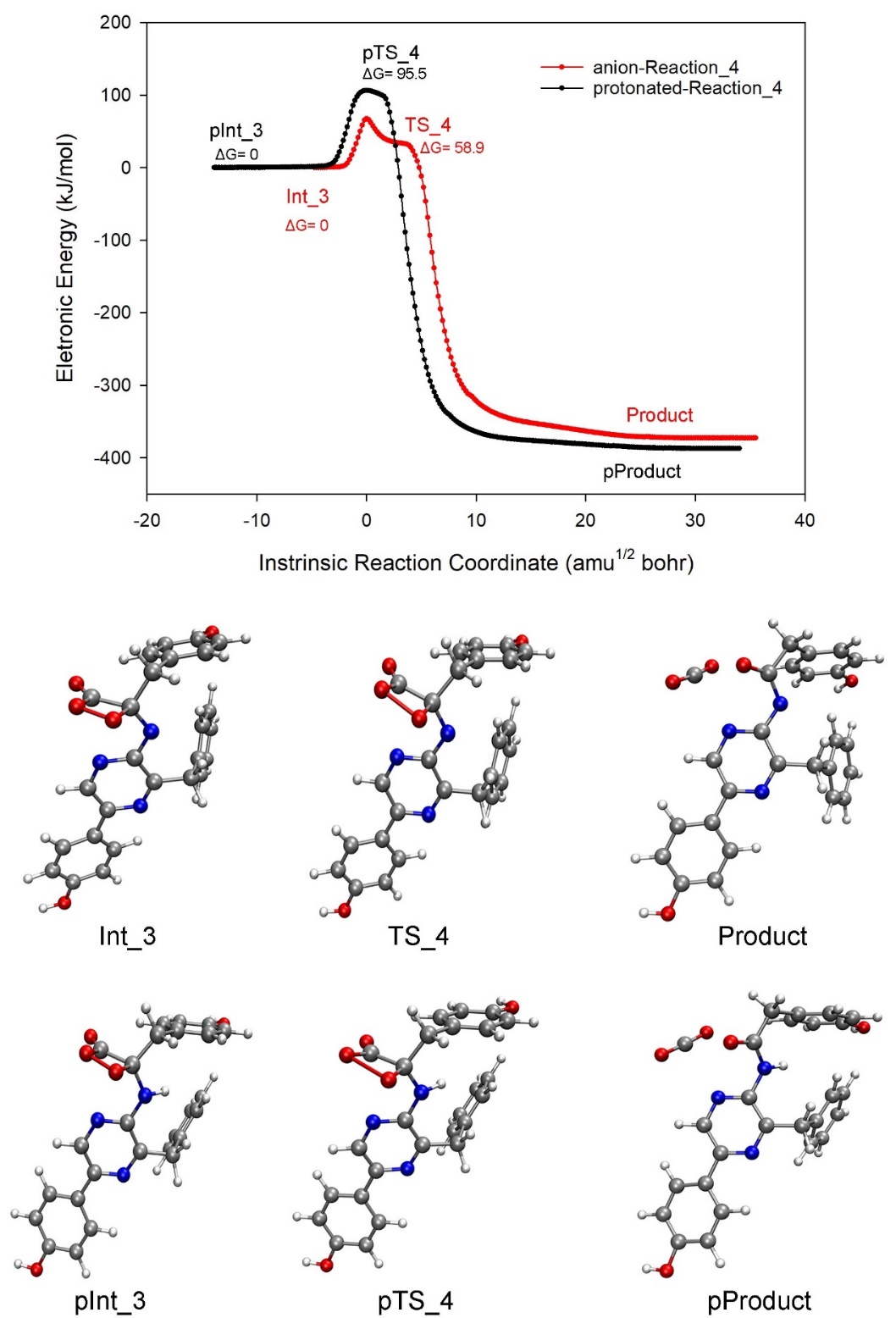


**S14 Fig.** The S0 PESs of the cleavage of isolated anionic/protonated dioxetanone intermediate (Reaction_4). The PESs for anionic and protonated Reaction_4 are depicted in red and black, respectively, with the structures at each stationary point displayed below.
